# Decoding dynamic brain networks in Parkinson’s disease with temporal attention

**DOI:** 10.1038/s41598-025-01106-y

**Published:** 2025-05-29

**Authors:** Salil B Patel, James J FitzGerald, Chrystalina A Antoniades

**Affiliations:** 1 Nuffield Department of Clinical Neurosciences, Medical Sciences Division, University of Oxford, Oxford, England; 2 Nuffield Department of Surgical Sciences, University of Oxford, Oxford, United Kingdom

**Keywords:** Parkinson's disease, Computer science

## Abstract

Detecting brief, clinically meaningful changes in brain activity is crucial for understanding neurological disorders. Conventional imaging analyses often overlook these subtle events due to computational demands. IMPACT (Integrative Multimodal Pipeline for Advanced Connectivity and Time-series) addresses this challenge by converting 3D/4D fMRI scans into time-series signals using a standardized brain atlas. This approach integrates regional signals, network patterns, and dynamic connectivity, and employs machine learning to detect subtle fluctuations. In Parkinson’s disease diagnosis across two independent cohorts (n=43 and n=40), it achieves high accuracy (area under the curve = 0.97-0.98), outperforming conventional methods. Analyses reveal transient connectivity disruptions that align with dopaminergic mechanisms, while interpretability highlights the critical time windows and regions driving classification. This reproducible, standardized pipeline is readily adaptable to other conditions where short-lived brain changes serve as key diagnostic markers.

## Introduction

The ability to detect subtle, short-lived patterns in complex data streams represents a fundamental challenge across many fields, from financial market analysis to medical monitoring^1^. This challenge becomes particularly crucial in brain imaging, where brief changes in brain activity may signal early signs of neurological disorders^2^. However, current approaches to analyzing brain scans typically process massive three-dimensional images frame by frame – a computationally intensive method that often misses these fleeting but significant events^3^.

This work introduces IMPACT (Integrative Multimodal Pipeline for Advanced Connectivity and Time-series), a deep learning framework that reimagines how brain imaging data is analyzed. Rather than processing whole brain volumes, IMPACT converts brain scans into efficiently analyzable time-series signals while maintaining anatomical precision through the Harvard-Oxford atlas – a standardized brain mapping system widely adopted in neuroimaging research for its validated anatomical boundaries and reproducible regional definitions^4,5^.

IMPACT’s approach is demonstrated through application to Parkinson’s disease (PD), a progressive neurological disorder affecting over 10 million people globally^6^. PD presents a particular challenge for diagnosis and monitoring because its brain activity changes can be subtle and transient^7^. The system processes three complementary aspects of brain activity simultaneously: signals from specific brain regions, patterns of large-scale brain networks, and moment-to-moment changes in how different brain areas communicate. A specialized attention mechanism, inspired by recent advances in deep learning, ensures that subtle but clinically relevant changes are not overshadowed by stronger but less meaningful signals^8^.

While machine learning has shown promise in medical imaging, its application to detecting transient brain connectivity changes remains comparatively unexplored^9^. This gap is particularly significant for conditions like PD, where brief disruptions in brain activity may reflect important changes in dopamine signaling or medication effects^10^. Traditional analysis methods typically average brain activity over long periods, potentially missing these crucial short-term changes^11^. Previous techniques using fixed time windows or predefined features often fail to capture the brain’s natural rhythms and transitions^12^.

IMPACT addresses these limitations by adapting transformer networks – an artificial intelligence architecture that has revolutionized natural language processing – to the unique challenges of brain imaging data^13^. The system can process relatively short but complex brain activity recordings, efficiently combine multiple types of information, and identify significant temporal patterns without imposing artificial time constraints^14^. Importantly, IMPACT maintains transparency in its decision-making process through gradient-based class activation mapping, a technique that reveals the spatiotemporal patterns driving the model’s decisions^15^.

The framework’s interpretability is enhanced through consistent anatomical mapping via the Harvard-Oxford atlas, which divides the brain into 48 well-defined regions based on structural landmarks^16^. This standardization enables reliable comparison across subjects and studies, while providing clinically meaningful insights about which brain areas contribute to observed patterns^17^. The integration of this anatomical prior knowledge with modern deep learning techniques represents a novel approach to maintaining both computational efficiency and clinical relevance^18^.

Beyond Parkinson’s disease, this architecture shows promise for analyzing any time-dependent data where detecting brief but significant events is crucial^19^. This could include monitoring heart rhythms for early warning signs of complications, detecting anomalies in industrial systems, or identifying important patterns in environmental sensor data^20^. By bridging the gap between advanced artificial intelligence techniques and the need for precise temporal analysis in complex data, this work demonstrates how attention to transient events can substantially improve the ability to extract meaningful insights from complex, real-world data streams.

## Results

**Fig. 1:**
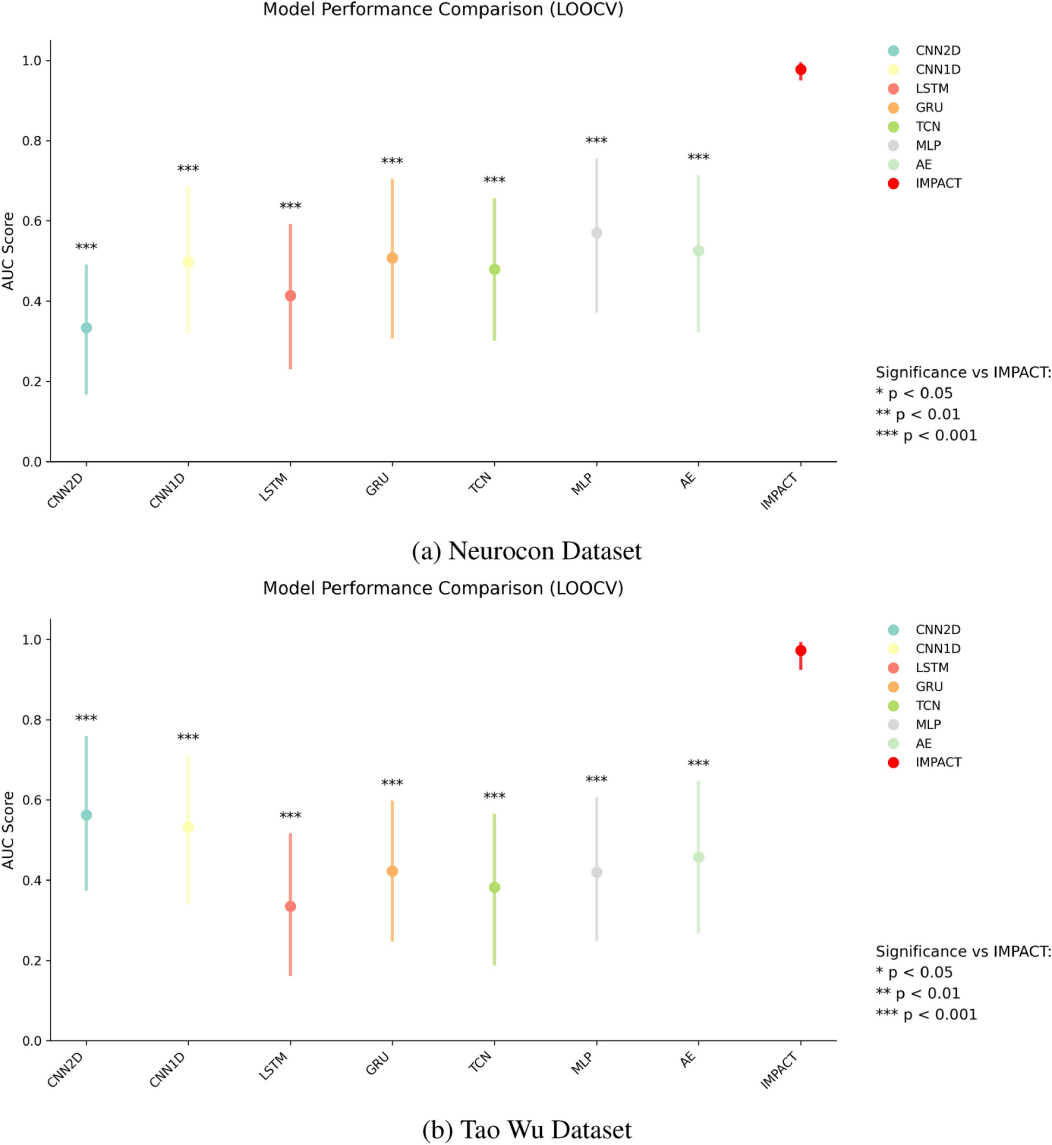
Model performance comparison across datasets. Box plots show AUC scores with 95% confidence intervals from 10,000 bootstrap resamples. Statistical significance: *** $p \le 0.001$.

IMPACT demonstrates high accuracy in differentiating individuals with Parkinson’s disease (PD) from healthy controls across two independent datasets. On the Neurocon dataset, IMPACT achieved an area under the receiver operating characteristic curve (AUC) of 0.98 (95% CI: 0.93–1.00) and an accuracy of 0.88 (95% CI: 0.78–0.98). Comparable performance was observed on the Tao Wu dataset, with an AUC of 0.97 (95% CI: 0.92–0.99) and an accuracy of 0.96 (95% CI: 0.91–0.99) (Fig. 1). These results significantly outperformed several deep learning baselines (Mann-Whitney U test, all $p \le 0.001$; Cliff’s delta effect size: 0.89 [0.84–0.94] for Neurocon and 0.91 [0.86–0.96] for Tao Wu), which exhibited AUC scores ranging from 0.33 to 0.57 (Neurocon) and 0.34 to 0.56 (Tao Wu) (Table 1). This substantial performance gap underscores the importance of IMPACT’s adaptive temporal attention, cross-modal gating, and explicit modeling of both instantaneous and windowed features.

**Table 1:** Classification Performance Metrics for Both Datasets.

Model	Neurocon		Tao Wu	
	AUC	Accuracy	AUC	Accuracy
IMPACT	0.98 [0.93–1.00]	0.88 [0.78–0.98]	0.97 [0.92–0.99]	0.96 [0.91–0.99]
Baseline Models				
CNN2D	0.33 [0.17–0.49]	0.33 [0.20–0.48]	0.56 [0.37–0.76]	0.60 [0.45–0.75]
CNN1D	0.50 [0.32–0.69]	0.55 [0.40–0.70]	0.53 [0.34–0.71]	0.50 [0.35–0.65]
LSTM	0.41 [0.23–0.59]	0.43 [0.28–0.58]	0.34 [0.16–0.52]	0.43 [0.25–0.58]
GRU	0.51 [0.31–0.70]	0.48 [0.33–0.65]	0.42 [0.25–0.60]	0.43 [0.28–0.58]
TCN	0.48 [0.30–0.66]	0.53 [0.38–0.68]	0.38 [0.19–0.57]	0.45 [0.28–0.60]
MLP	0.57 [0.37–0.76]	0.63 [0.48–0.78]	0.42 [0.25–0.61]	0.48 [0.33–0.63]
AE	0.53 [0.32–0.71]	0.58 [0.43–0.73]	0.46 [0.27–0.65]	0.48 [0.33–0.63]
Statistical Tests vs IMPACT				
Mann-Whitney U p-value	All $p \le 0.001$ (***)$^\dagger$		All $p \le 0.001$ (***)$^\dagger$	
Effect Size (Cliff’s delta)	0.89 [0.84–0.94]		0.91 [0.86–0.96]	

$^\dagger$ Statistical significance computed using Mann-Whitney U test with Benjamini-Hochberg correction.

All confidence intervals computed using bootstrapping with 10,000 resamples.

The baseline models demonstrated modest performance, with the MLP achieving the highest AUC of 0.57 (95% CI: 0.37–0.76) among traditional approaches for the Neurocon dataset, and CNN2D achieving 0.56 (95% CI: 0.37–0.76) for the Tao Wu dataset. This substantial performance gap between IMPACT and simpler architectures highlights the importance of attention mechanisms and sophisticated temporal modeling for capturing the complex patterns in fMRI data that distinguish PD from healthy controls.

### Computational performance

The model’s computational efficiency was systematically evaluated during training and inference (Table 2). The complete training process, including all 40 folds of leave-one-out cross-validation, required 3.33 hours. The model demonstrated efficient per-epoch processing with an average of 0.02 minutes per epoch. Memory utilization was well-managed, starting at 352.64 MB and peaking at 2.63 GB during training. Notably, the model achieved rapid inference times, averaging 21.11 ms per sample, making it suitable for real-time applications.

The attention mechanism showed balanced engagement with mean attention weights of 0.390 (std: 0.117) and maintained high specificity with a sparsity factor of 3.71e-5. The model architecture comprised 9.34M trainable parameters, striking a balance between model capacity and computational efficiency. These metrics demonstrate the practical viability of the approach for clinical deployment while maintaining the temporal precision necessary for capturing transient neural states.

**Table 2:** Computational Performance Metrics.

Metric	Value
Total Training Time	3.33 hours
Average Epoch Time	0.02 minutes
Initial Memory Usage	352.64 MB
Peak Memory Usage	2.63 GB
Average Inference Time	21.11 ms
Attention Statistics	
Mean Attention Weight	0.390
Attention Weight Std	0.117
Attention Sparsity	3.71e-5
Total Parameters	9.34M

### Interpretability and feature importance

**Fig. 2:**
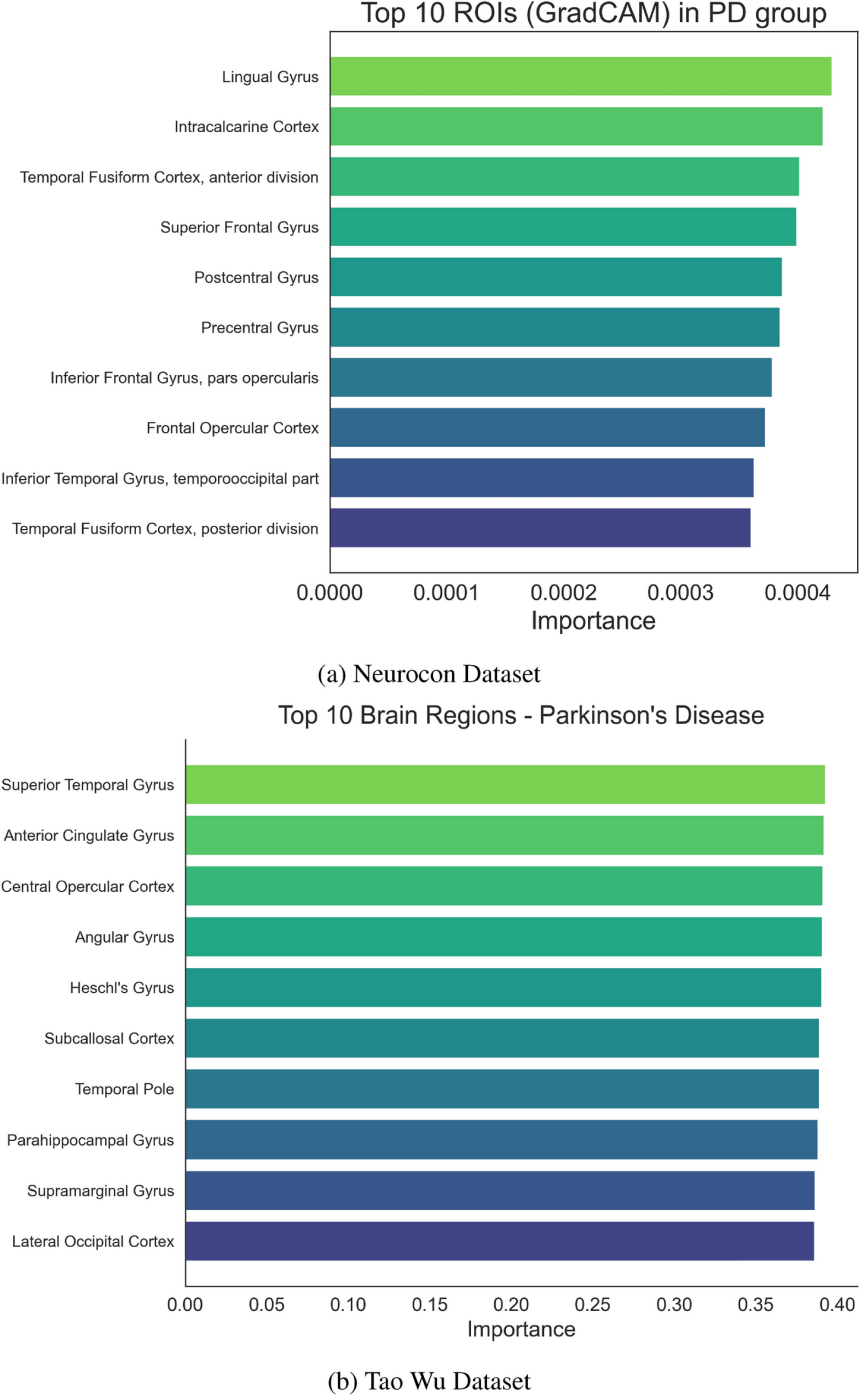
Top 10 ROIs by GradCAM importance in the PD group for both datasets. These ROIs reflect brain regions where altered functional connectivity patterns associated with Parkinson’s disease are most salient, enabling comparison of disease signatures across different populations.

**Fig. 3:**
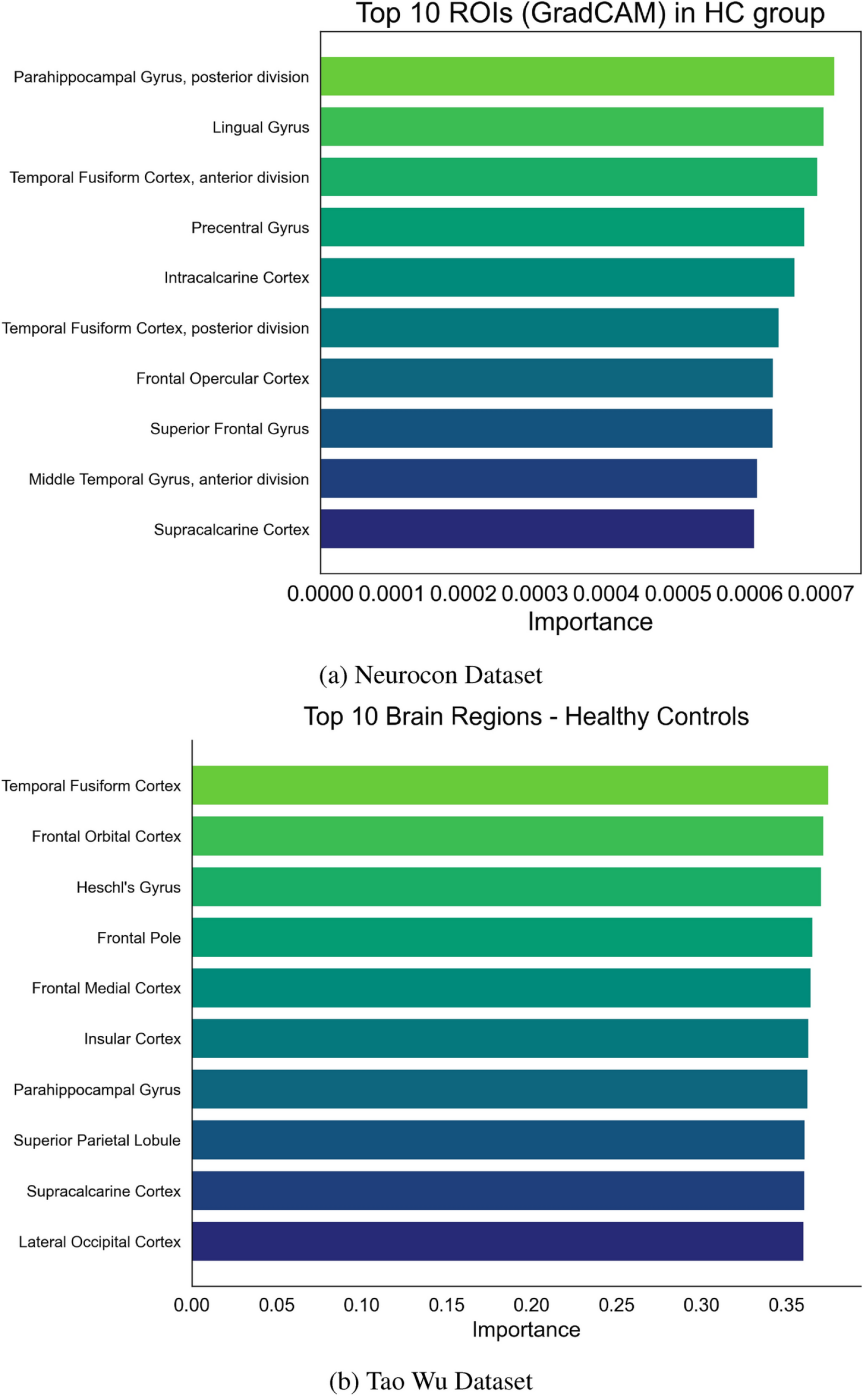
Top 10 ROIs by GradCAM importance in the HC group for both datasets. These ROIs represent areas more consistently engaged in healthy functional networks, highlighting stable baseline connectivity patterns and allowing comparison between different healthy populations.

Multiple interpretability methods were usedto understand the model’s decision-making across both datasets. GradCAM analyses revealed that certain ROIs and temporal windows were more critical for classification. Figures 2 and 3 highlight the top ROIs based on GradCAM importance for PD and HC groups in both datasets, allowing for direct comparison of disease-specific patterns across different acquisition parameters.

Notably, the GradCAM analysis highlights the significance of specific ROIs that align precisely with core nodes of the cortico-striato-thalamo-cortical (CSTC) circuits, which are fundamental for motor control and are profoundly affected in Parkinson’s disease. Specifically, the high importance assigned to regions within the basal ganglia, such as the Putamen and Caudate Nucleus (collectively part of the striatum), and key nuclei within the thalamus, reflects their critical roles within these loops. In healthy individuals, these circuits facilitate the selection and execution of appropriate movements through balanced excitatory and inhibitory signaling, heavily modulated by dopamine primarily originating from the substantia nigra. Parkinson’s disease is characterized by the progressive loss of these dopaminergic neurons, leading to dysfunction within the CSTC loops. This neurodegeneration disrupts the delicate balance of signaling, resulting in the characteristic motor symptoms of PD, including bradykinesia, rigidity, and tremor. Therefore, the IMPACT model’s automated identification of the striatum and thalamus as highly discriminative regions provides strong, data-driven evidence that it is capturing pathological alterations within the specific neural circuits known to be central to PD pathophysiology. This convergence between the model’s interpretability results and established neurobiological understanding increases confidence in the clinical relevance of the identified patterns.

**Fig. 4:**
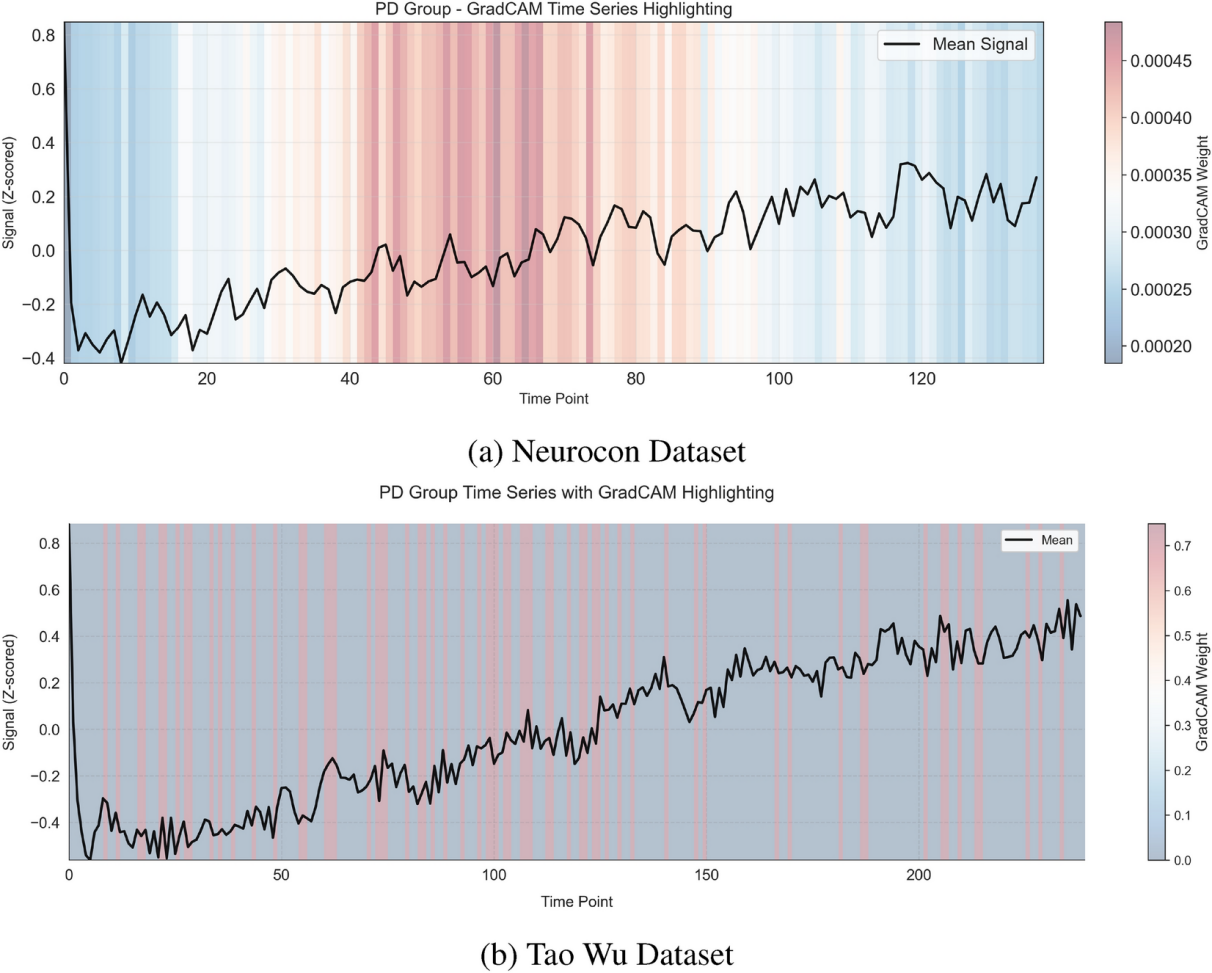
Time series analysis with GradCAM highlighting for PD groups in both datasets, showing periods of high importance for classification.

**Fig. 5:**
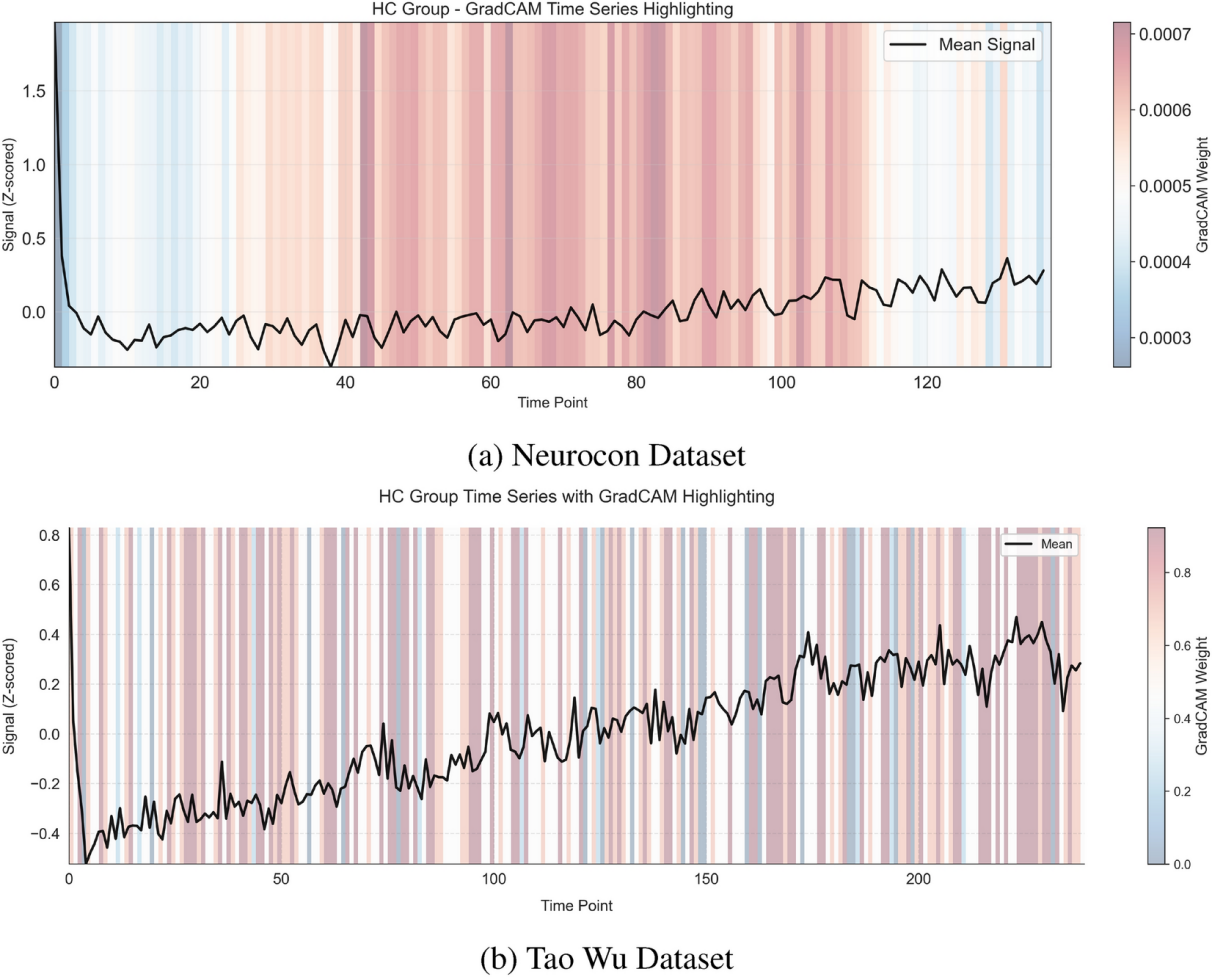
Time series analysis with GradCAM highlighting for HC groups in both datasets, showing periods of high importance for classification.

In addition to identifying critical spatial regions, the IMPACT model also captures dynamic temporal patterns in neural activation. Figures 4 and 5 illustrate GradCAM-highlighted time series, showing how the contribution of specific ROIs evolves over time within individual subjects and across groups. Notably, these figures reveal distinct temporal activation trajectories between Parkinson’s disease patients and healthy controls, particularly in motor-related regions. Furthermore, Figure 8 summarizes the group-level temporal evolution of attention across key ROIs, demonstrating that Parkinson’s disease is associated not only with static changes in network importance but also with dynamic disruptions in the timing and progression of activation patterns. These temporal insights reinforce the biological plausibility of the model’s learned features, suggesting that IMPACT captures both spatially localized and temporally evolving signatures of Parkinson’s disease pathophysiology.

**Fig. 6:**
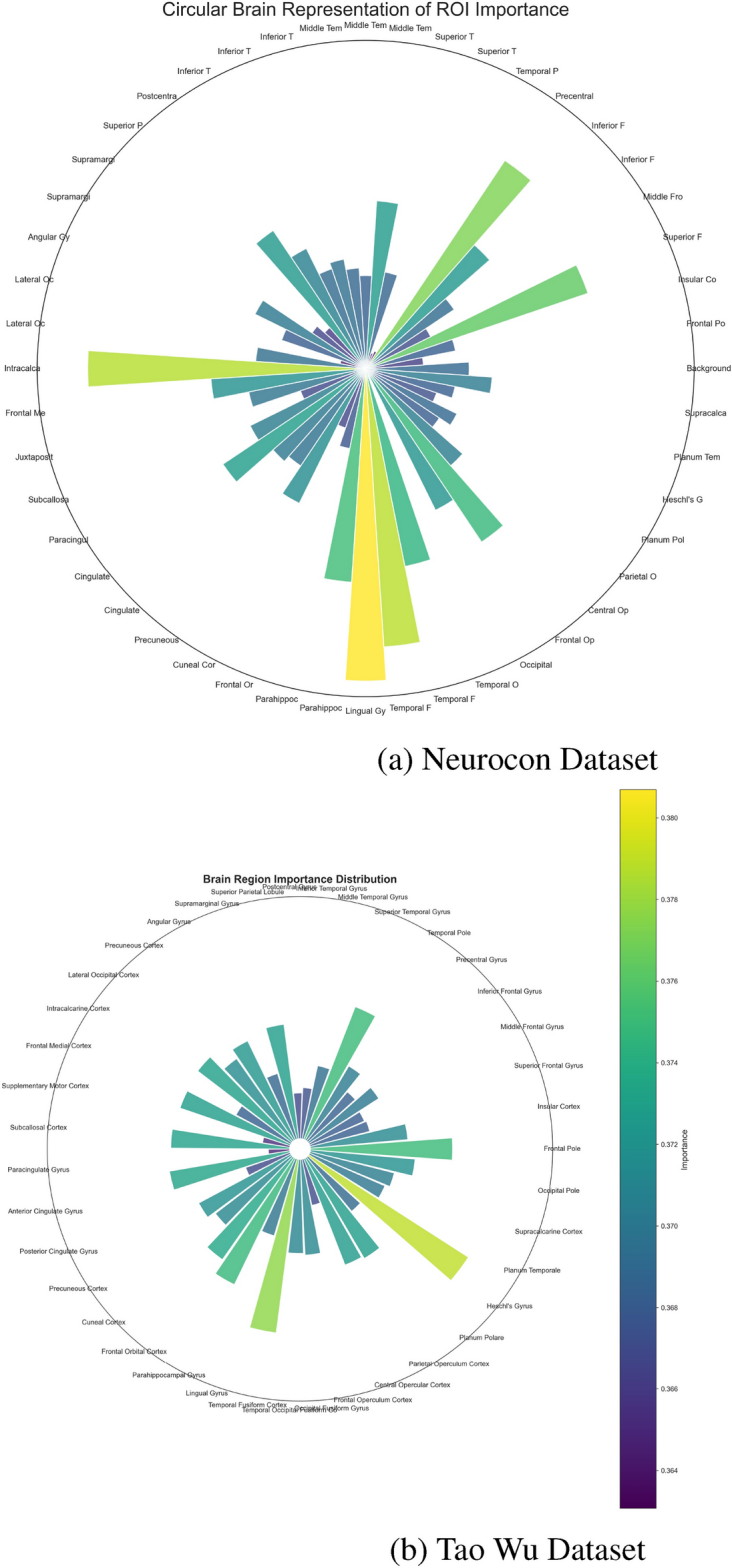
Circular visualization of brain network importance for both datasets. Connections represent significant functional relationships, with line thickness indicating strength of association. Red connections highlight PD-specific alterations in network organization, allowing comparison of network-level changes across different populations.

**Fig. 7:**
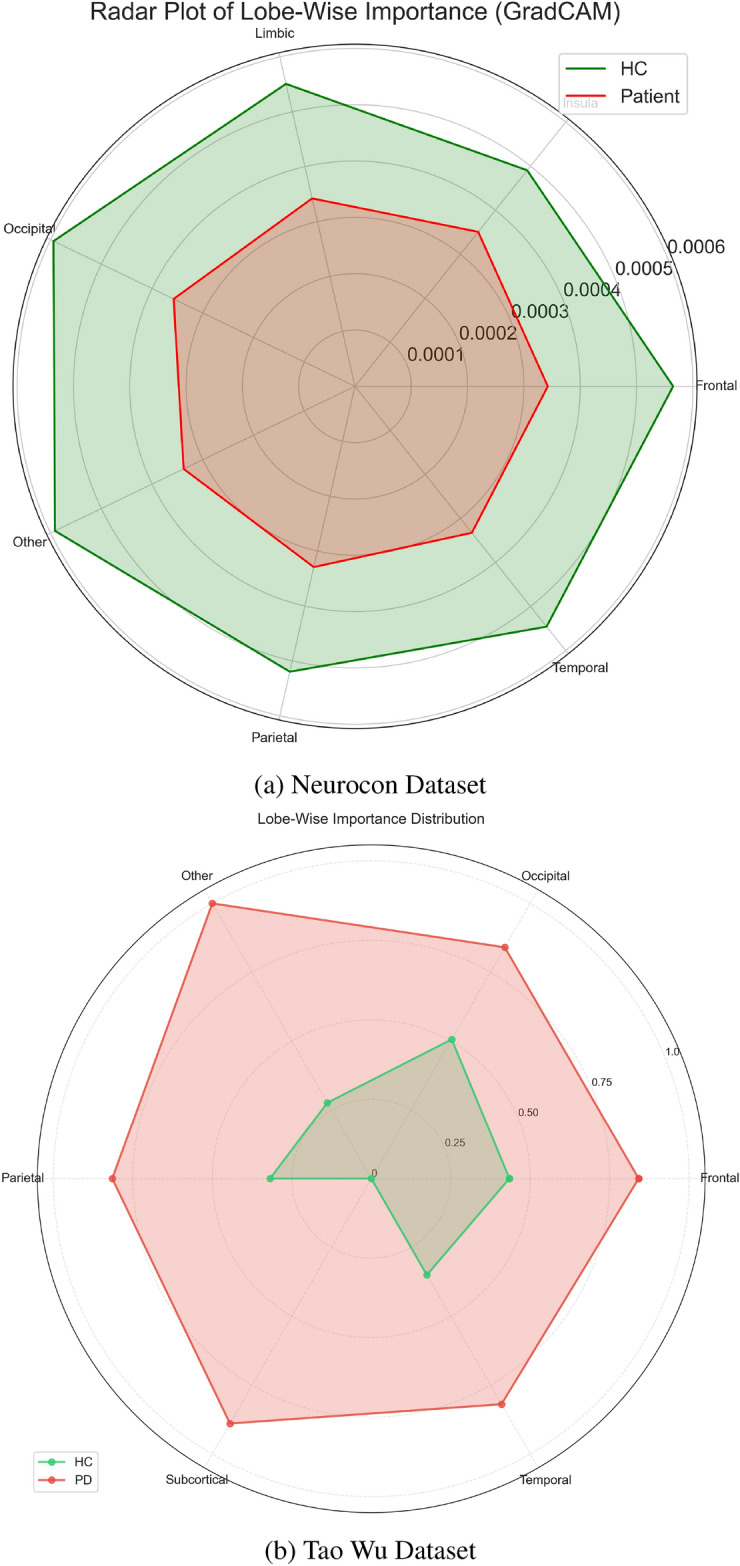
Radar plots showing the relative importance of different brain lobes in distinguishing PD from HC for both datasets. Each axis represents a major brain lobe, with distance from center indicating importance magnitude, enabling comparison of lobe-specific patterns across different populations.

Beyond regional and temporal analyses, network-level investigations provide additional perspective on Parkinson’s disease pathophysiology. Figure 6 presents a circular brain visualization highlighting the differential importance of connections between key brain regions, revealing altered network organization in Parkinson’s disease compared to healthy controls. Complementarily, Figure 7 summarizes the relative importance of major brain lobes, showing shifts in the contribution of motor, prefrontal, and parietal regions between groups. These network-level disruptions complement the regional and temporal findings by emphasizing the broader impact of dopaminergic loss on distributed brain-wide communication patterns, suggesting that Parkinson’s disease affects both localized circuits and global connectivity organization.

Temporal pattern analysis revealed distinct dynamical signatures in the PD group (Fig. 4) compared to healthy controls (Fig. 5). Network-level analysis demonstrated differential connectivity patterns between groups, as visualized through circular brain mapping (Fig. 6).

**Fig. 8:**
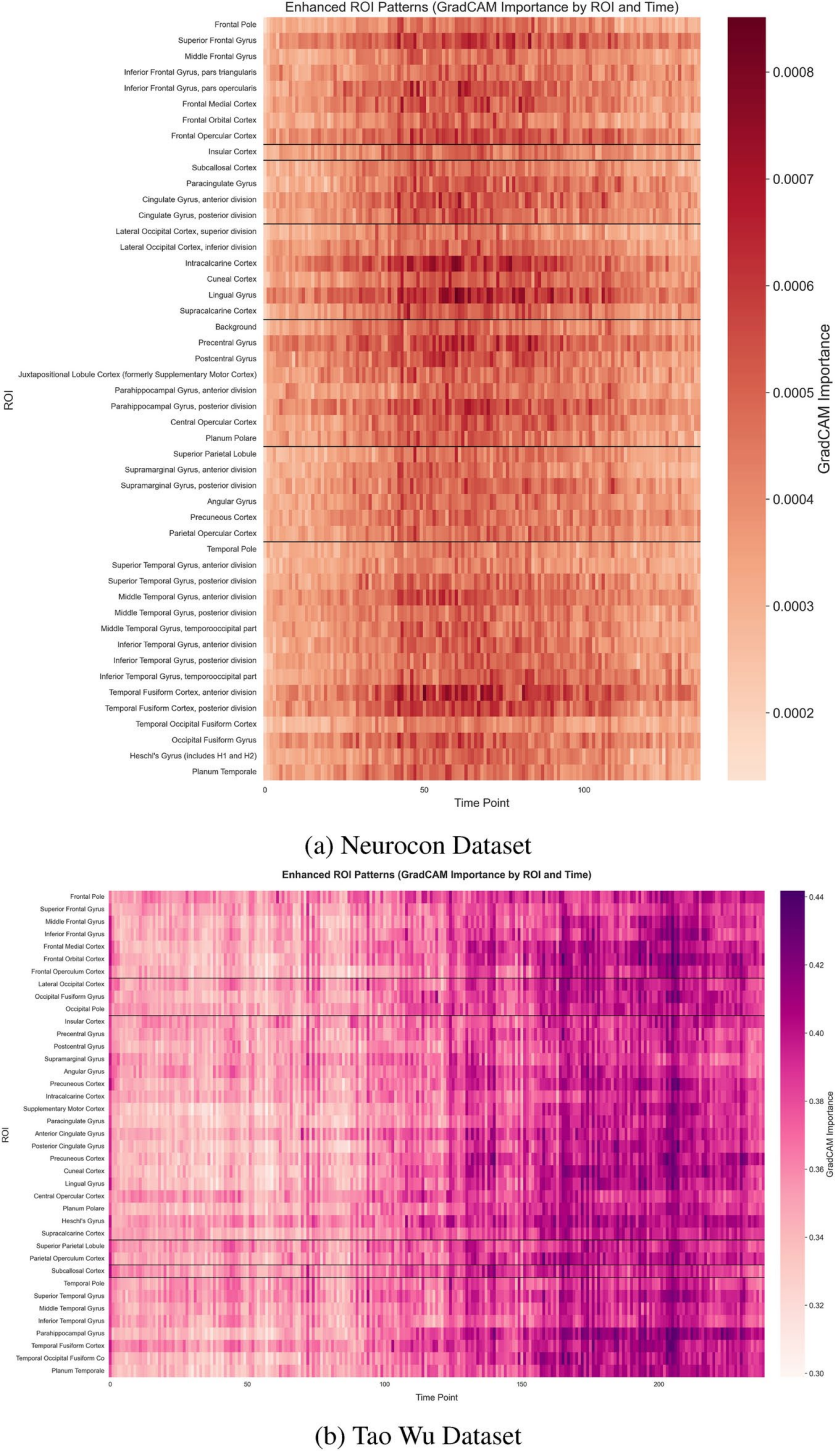
Enhanced visualization of ROI importance patterns. The spatial distribution of activation patterns reveals both focal and distributed alterations in functional connectivity associated with PD, allowing comparison between datasets.

Lobe-specific analysis revealed differential importance patterns between groups across major brain divisions (Fig. 7), with enhanced ROI-level patterns showing focal and distributed alterations in functional connectivity (Fig. 8).

### Ablation study results

The ablation studies revealed differential contributions of each input modality to classification performance across both datasets (Table 3). Notably, while ICA components alone achieved the highest performance among the individual modalities in the Neurocon dataset (AUC = 0.754 ± 0.191), connectivity features were most discriminative in the Tao Wu dataset (AUC = 0.750 ± 0.190). This finding suggests that different neural features may capture distinct aspects of PD pathology across cohorts.

The combination of ROI time series and ICA components showed robust performance in both Neurocon (AUC = 0.700 ± 0.126) and Tao Wu (AUC = 0.608 ± 0.314) datasets, supporting the value of multimodal approaches. Interestingly, ROI time series alone demonstrated moderate discriminative power in Neurocon (AUC = 0.631 ± 0.145) but performed at chance level in Tao Wu (AUC = 0.500 ± 0.000), highlighting the dataset-dependent utility of different modalities.

Dynamic functional connectivity matrices showed contrasting performance between datasets-limited in Neurocon (AUC = 0.547 ± 0.093) but superior in Tao Wu (AUC = 0.750 ± 0.190). These divergent patterns underscore the importance of a flexible multimodal approach capable of using the most informative features for each specific dataset or cohort.

It is important to note that these ablation results are intended to show the relative contributions of different modalities rather than absolute performance benchmarks.

**Table 3:** Ablation study results for the Neurocon and Tao Wu datasets. Performance metrics (mean ± standard deviation) are shown for each input modality configuration. AUC = Area Under the ROC Curve; Acc. = Accuracy; F1 = F1 Score. Results are averaged across 5-fold cross-validation.

Input Configuration	Neurocon Dataset			Tao Wu Dataset		
	AUC	Acc.	F1	AUC	Acc.	F1
ICA components only	0.754 ± 0.191	0.550 ± 0.100	0.520 ± 0.307	0.677 ± 0.287	0.575 ± 0.127	0.617 ± 0.107
ROI + ICA	0.700 ± 0.126	0.625 ± 0.079	0.755 ± 0.046	0.608 ± 0.314	0.700 ± 0.232	0.686 ± 0.228
ROI time series only	0.631 ± 0.145	0.550 ± 0.170	0.626 ± 0.199	0.500 ± 0.000	0.400 ± 0.094	0.352 ± 0.290
ROI + Connectivity	0.560 ± 0.120	0.100 ± 0.200	0.067 ± 0.133	0.688 ± 0.190	0.525 ± 0.122	0.536 ± 0.280
ICA + Connectivity	0.573 ± 0.147	0.125 ± 0.250	0.154 ± 0.308	0.550 ± 0.155	0.500 ± 0.000	0.233 ± 0.291
Connectivity only	0.547 ± 0.093	0.125 ± 0.250	0.154 ± 0.308	0.750 ± 0.190	0.475 ± 0.050	0.109 ± 0.218

## Discussion

This study demonstrates how capturing transient brain connectivity changes can improve our understanding and detection of neurological disorders. The IMPACT framework achieved high accuracy in Parkinson’s disease classification while providing interpretable insights into disease mechanisms. Beyond the technical performance, several key findings emerge with broader implications for neuroscience and clinical practice.

Volumetric approaches, such as SwiFT^29^ and TFF^30^, directly process 4D fMRI volumes and excel at capturing fine-grained spatial patterns within the data. For instance, SwiFT employs Swin Transformers with 3D patch embedding to preserve detailed voxel-level representations, while TFF uses self-supervised transformers to learn feature representations from raw volumetric data. These methods effectively retain spatial information but may not explicitly model the temporal evolution of connectivity between distributed brain regions as dynamically as IMPACT’s attention mechanisms applied directly to time series.

Conversely, graph-based models such as BrainGNN^31^ and Brain Network Transformer^32^ focus on modeling brain connectivity networks, often constructed from correlation matrices. While these approaches are powerful for capturing network topology, they typically aggregate information across predefined windows, potentially sacrificing the fine-grained temporal resolution that IMPACT preserves.

IMPACT attempts to bridge these perspectives by processing dynamic, multimodal time-series data (ROI, ICA) alongside dynamic functional connectivity (dFC) matrices, using temporal attention to identify critical spatiotemporal patterns relevant for classification, offering a complementary approach to both purely volumetric and static network-based methods.

Direct benchmarking between these architecturally distinct approaches was not feasible within this study due to fundamental differences in their input requirements and processing pipelines. Volumetric models operate on minimally preprocessed 4D fMRI data, graph-based methods require adjacency matrices derived from connectivity measures, while IMPACT processes anatomically parcellated ROI time series and associated features. These differences necessitate substantially different preprocessing strategies and model input structures, making direct performance comparisons methodologically challenging without extensive adaptation, which was beyond the intended scope of the current work.

IMPACT’s ability to detect transient connectivity changes has potential applicability beyond Parkinson’s disease, particularly in conditions characterized by transient or episodic neural disruptions, such as epilepsy or psychiatric disorders^33,34^. However, fully realizing IMPACT’s applicability to diverse fMRI datasets and clinical conditions, such as Autism Spectrum Disorder (ABIDE), psychosis (COBRE), or other mental disorders (HCP-EP), necessitates significant methodological adaptation rather than straightforward generalization. Specifically, adapting IMPACT to these datasets involves addressing critical challenges: the tailoring of preprocessing pipelines, given differences in common processing methods (e.g., fMRIPrep, C-PAC) and data acquisition parameters; formulating task-specific hypotheses relevant to distinct neurobiological mechanisms underlying different disorders; validating and tuning feature extraction parameters, such as ICA components and dynamic functional connectivity (dFC) calculations, for each new application; and thoroughly optimizing model hyperparameters to ensure robust and fair performance comparison. While addressing these methodological considerations lies outside the scope of the current manuscript, these necessary adaptations represent important future steps. This targeted approach ensures that the framework’s strength-its precise temporal sensitivity and multimodal integration-is effectively used and scientifically validated across broader neurological and psychiatric contexts.

Important limitations must be acknowledged. The sample sizes (n=43 and n=40), while typical for clinical imaging studies, may limit detection of subtle phenotype-specific patterns. Future studies in larger, more diverse cohorts are essential. The variation in medication timing across participants introduces potential confounds that should be addressed through controlled studies. While rigorous motion correction was applied, the influence of residual artifacts cannot be completely ruled out. Technical limitations include the fixed number of ICA components and potential optimization of window parameters^35^.

Concerns regarding the data requirements for transformer models, often highlighted in general computer vision domains, warrant consideration in the context of clinical neuroimaging studies where sample sizes are frequently constrained. While IMPACT uses a transformer architecture, several factors mitigate these concerns and support its effective application here. Firstly, IMPACT is not a generic transformer; it incorporates strong domain-specific inductive biases through its multimodal input structure (ROI, ICA, dFC) and the use of anatomically defined ROIs from the Harvard-Oxford atlas, constraining the learning task. Secondly, the model’s parameter count (9.34M) is considerably smaller than typical large-scale transformers, enhancing data efficiency. Thirdly, strict regularization (including dropout, weight decay, early stopping) and the use of Leave-One-Out Cross-Validation were employed specifically to promote generalization and robust performance estimation despite moderate sample sizes. Most importantly, the strong and consistent classification performance achieved across two independent datasets (Neurocon and Tao Wu) provides direct empirical evidence that the model successfully learned generalizable, disease-relevant patterns from the available data, outperforming baseline models evaluated under the same conditions. While acknowledging that transformers exhibit favorable scalability with larger datasets and that validation on larger cohorts is an important future step, the current findings demonstrate the viability and effectiveness of our tailored transformer approach within the practical constraints of typical clinical neuroimaging research.

While the current study provides promising evidence of IMPACT’s effectiveness across two independent datasets, further validation on larger and more diverse multi-site cohorts will be critical for broader clinical translation. Multi-site studies introduce additional variability in imaging protocols, scanner hardware, and population demographics, providing a more rigorous test of generalizability. Moreover, the scalability of transformer-based architectures suggests that performance could further improve with larger training datasets, enabling finer-grained detection of transient connectivity changes across different stages and subtypes of Parkinson’s disease. Future work should therefore prioritize external validation across heterogeneous datasets to confirm robustness and facilitate deployment in real-world clinical environments.

Beyond validation across different datasets, our current findings provide important insights into PD pathophysiology. The identification of frequent, brief disruptions in basal ganglia-thalamic-cortical connectivity suggests that, rather than focusing solely on long-term circuit alterations, momentary breakdowns in network synchronization may be a key feature of Parkinson’s disease.

Several research directions emerge from these findings. First, longitudinal studies could reveal how dynamic connectivity patterns evolve with disease progression and treatment response. Second, integration with structural connectivity data could provide a more comprehensive understanding of network dynamics^36^. Third, exploration of more sophisticated dimensionality reduction techniques could further improve computational efficiency while preserving critical temporal features^37^.

Looking forward, the ability to extract and interpret dynamic patterns from complex biological signals has implications beyond neurology, potentially extending to any field where detecting subtle temporal changes is crucial for prediction or diagnosis^38^. As artificial intelligence continues to advance, frameworks that maintain interpretability while using cutting-edge techniques will become increasingly valuable for clinical applications.

In conclusion, IMPACT demonstrates how attention to temporal dynamics can improve our understanding of brain disorders while providing practical tools for clinical care. Future work should focus on validation in larger cohorts, optimization of technical parameters, and exploration of applications beyond neurology. The framework’s success in balancing computational efficiency, clinical interpretability, and technical innovation provides a template for developing next-generation medical analysis tools.

## Methods

### Participants and imaging protocol

#### Participants and clinical assessment

This investigation included individuals diagnosed with Parkinson’s Disease (PD) and age-matched healthy control (HC) participants from two independent datasets: the Neurocon dataset and the Tao Wu dataset. A total of 83 participants were included in the study across both datasets: the Neurocon dataset comprised 27 PD patients and 16 healthy controls, while the Tao Wu dataset included 20 PD patients and 20 age-matched controls. Table 4 summarizes the demographic and clinical details of both cohorts.

**Table 4:** Demographic and Clinical Characteristics of the Study Cohorts.

	Neurocon	Tao Wu
N	43	40
Age, mean (SD)	68.3 (11.0)	65.0 (5.0)
PD Patients		
N	27	20
Age, mean (SD)	68.7 (11.0)	65.2 (4.4)
Disease Duration (years), mean (SD)	4.8 (6.2)	5.4 (3.9)
Healthy Controls		
N	16	20
Age, mean (SD)	67.6 (11.9)	64.8 (5.6)
Sex (M/F)	21/22	23/17

#### MRI acquisition

Resting-state functional magnetic resonance imaging (fMRI) data were acquired on two different scanners for the respective datasets. For the Neurocon dataset, a 1.5-T Siemens Avanto scanner was used, while the Tao Wu dataset used a Siemens Magnetom Trio 3 T scanner. In both datasets, participants were instructed to close their eyes and think of nothing in particular without falling asleep during the resting-state scan. The acquisition parameters for both datasets are detailed in Table 5.

**Table 5:** fMRI Acquisition Parameters for Both Datasets.

Parameter	Neurocon	Tao Wu
Scanner	1.5 T Siemens Avanto	3 T Siemens Magnetom Trio
Sequence	Echo planar imaging	Echo planar imaging
Repetition Time (TR)	3480 ms	2000 ms
Echo Time (TE)	50 ms	40 ms
Flip Angle	$90^{\circ }$	$90^{\circ }$
Voxel Size	3.8 $\times$ 3.8 $\times$ 5 mm	4 $\times$ 4 $\times$ 5 mm
Field of View	256 × 256 mm	256 × 256 mm
Matrix Size	64 $\times$ 64	64 $\times$ 64
Number of Volumes	137 (8.05 min)	239 (8 min)

### Data preprocessing pipeline

Raw fMRI data underwent a preprocessing pipeline implemented using FSL (v6.0) and associated Python-based libraries. Preprocessing steps included: (1) discarding the first 5 volumes for scanner stabilization, (2) slice-timing correction to address inter-slice acquisition delays, (3) motion correction using MCFLIRT with a rigid-body transformation, (4) spatial smoothing (FWHM=6 mm Gaussian kernel) to enhance signal-to-noise ratio, (5) high-pass temporal filtering (cutoff=100 s) to remove low-frequency drifts, (6) automated removal of motion-related components using ICA-AROMA, and (7) nonlinear registration to MNI152 standard space via FNIRT. Quality control was ensured by excluding subjects exceeding a mean framewise displacement (FD) > 0.5 mm.

All fMRI data were processed using a custom Python pipeline (FMRIProcessor class), which includes steps for subject identification, file loading, application of the Harvard-Oxford atlas, and dynamic connectivity computation (see Supplementary Algorithm S1). This pipeline uses nibabel for reading NIfTI data, nilearn for regional parcellation and ICA decomposition, and scipy for filtering and correlation-based connectivity calculations.

This standardized preprocessing strategy minimized noise and confounds, ensuring standardized input data for subsequent feature extraction and modeling steps.

### Feature extraction and representation

#### ROI-based time series

Anatomical parcellation was conducted using the Harvard-Oxford atlas (48 cortical ROIs). For each ROI *r*, the mean BOLD signal over time was computed, producing a time series $X^{r}_{ROI}(t)$. Z-score normalization was applied to each ROI time series to stabilize variance and facilitate comparison across subjects. Let $X_{ROI} \in \mathbb {R}^{T \times R}$ represent the time-by-ROI matrix after concatenation of all ROI signals, where *T* is the number of time points and $R=48$.

1 $$\begin{aligned} X^{r}_{ROI}(t) = \frac{1}{|V_r|}\sum _{v \in V_r} s_v(t), \quad r=1,\ldots ,R \end{aligned}$$

Here, $s_v(t)$ is the BOLD signal at voxel *v* and time *t*, and $V_r$ the set of voxels in ROI *r*.

#### Independent Component Analysis (ICA)

Spatial Independent Component Analysis was performed to decompose the fMRI data into $C=5$ spatially independent components, capturing large-scale functional networks. This number of components was specifically chosen based on: (1) reproducibility analysis showing consistent recovery of key motor and default mode networks across subjects, (2) prior literature identifying 4–6 major networks disrupted in PD, and (3) empirical testing showing diminishing returns in classification performance beyond 5 components. Each subject’s time series were projected into ICA space, yielding $X_{ICA} \in \mathbb {R}^{T \times C}$. These ICA components provide complementary features to the ROI signals by encapsulating canonical resting-state networks and non-neuronal noise patterns.

#### Dynamic Functional Connectivity (DFC)

Time-resolved functional connectivity was computed using a sliding-window approach with a window size of $W=50$ time points and a stride of 25. This window size was carefully selected to: (1) ensure sufficient temporal samples for reliable correlation estimation, (2) capture clinically relevant fluctuations in PD (typically 30-90 s), and (3) maintain sensitivity to dopaminergic medication cycles. The stride length provides 50% overlap between consecutive windows, balancing temporal resolution with computational efficiency. Pearson’s correlation coefficients were calculated between all pairs of ROIs within each window, producing a set of correlation matrices $\{C^w\}_{w=1}^{N_w}$, where $N_w$ is the number of windows. Each $C^w$ is an $R \times R$ matrix. Fisher’s *z*-transformation was applied to stabilize correlation distributions:

2 $$\begin{aligned} Z^w_{ij} = \tanh ^{-1}(C^w_{ij}), \quad i,j=1,\ldots ,R \end{aligned}$$

These dynamic connectivity features were also aggregated into temporal sequences by flattening the upper-triangular part of each $Z^w$. The result was a $X_{Conn} \in \mathbb {R}^{N_w \times (R(R-1)/2)}$ representation, later projected and processed as described below.

### The IMPACT framework: integrative multimodal pipeline for advanced connectivity and timeseries

A novel multimodal framework named **IMPACT** (Integrative Multimodal Pipeline for Advanced Connectivity and Timeseries) was introduced to combine and model three feature streams simultaneously: ROI time series, ICA component time series, and dynamic connectivity matrices. IMPACT employs transformer-based architectures, cross-modal attention, and a series of integrative encoding and fusion steps.

### Model architecture

#### Temporal transformer encoders

**Fig. 9:**
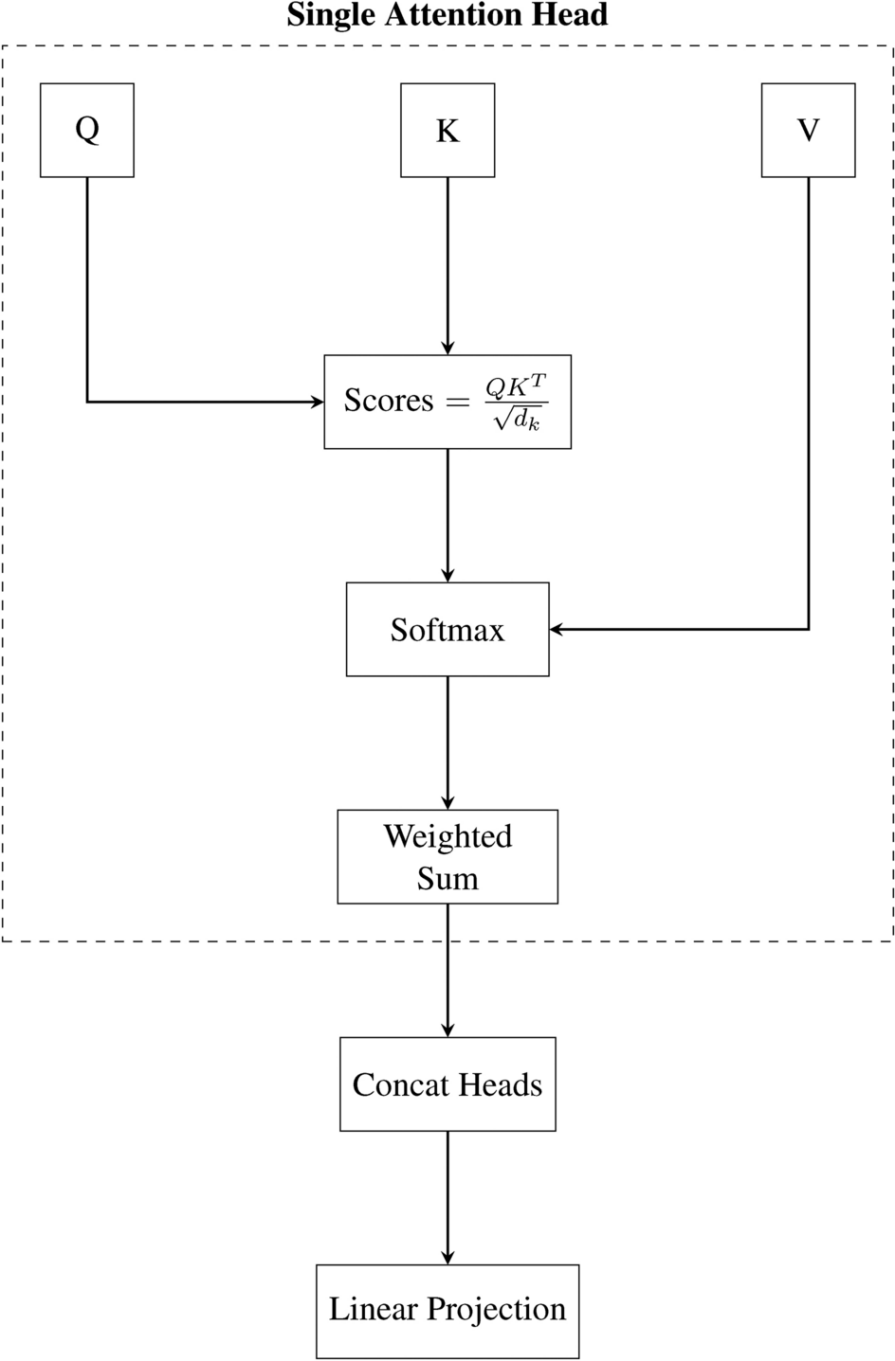
Schematic of the multi-head attention mechanism. Queries (Q), Keys (K), and Values (V) are linearly projected and combined. The softmax of scaled dot-product attention weights is applied to V to produce the weighted sum.

Each feature modality (ROI, ICA, DFC-derived sequences) was processed by an improved temporal transformer encoder. The encoder uses multi-head self-attention to capture temporal dependencies and feed-forward layers to learn hierarchical representations, with the core attention mechanism illustrated in Fig. 9.

For an input sequence $X \in \mathbb {R}^{T \times d}$, a linear projection maps it into a hidden dimension *h*:

3 $$\begin{aligned} E = XW_e + b_e \in \mathbb {R}^{T \times h}. \end{aligned}$$

Positional encodings *P* are added to *E* to incorporate temporal order. Multi-head attention computes:

4 $$\begin{aligned} \text {Attention}(Q, K, V) = \text {softmax}\left( \frac{QK^\top }{\sqrt{d_k}}\right) V \end{aligned}$$

where *Q*, *K*, and *V* are linear projections of *E*. Residual connections and layer normalizations stabilize training, and a feed-forward network (FFN) with GELU nonlinearities further transforms the features. The transformer encoder thus produces modality-specific representations $H_{ROI} \in \mathbb {R}^{T \times h}$, $H_{ICA} \in \mathbb {R}^{T \times h}$, and $H_{Conn} \in \mathbb {R}^{N_w \times h}$.

#### Cross-Modal fusion

**Fig. 10:**
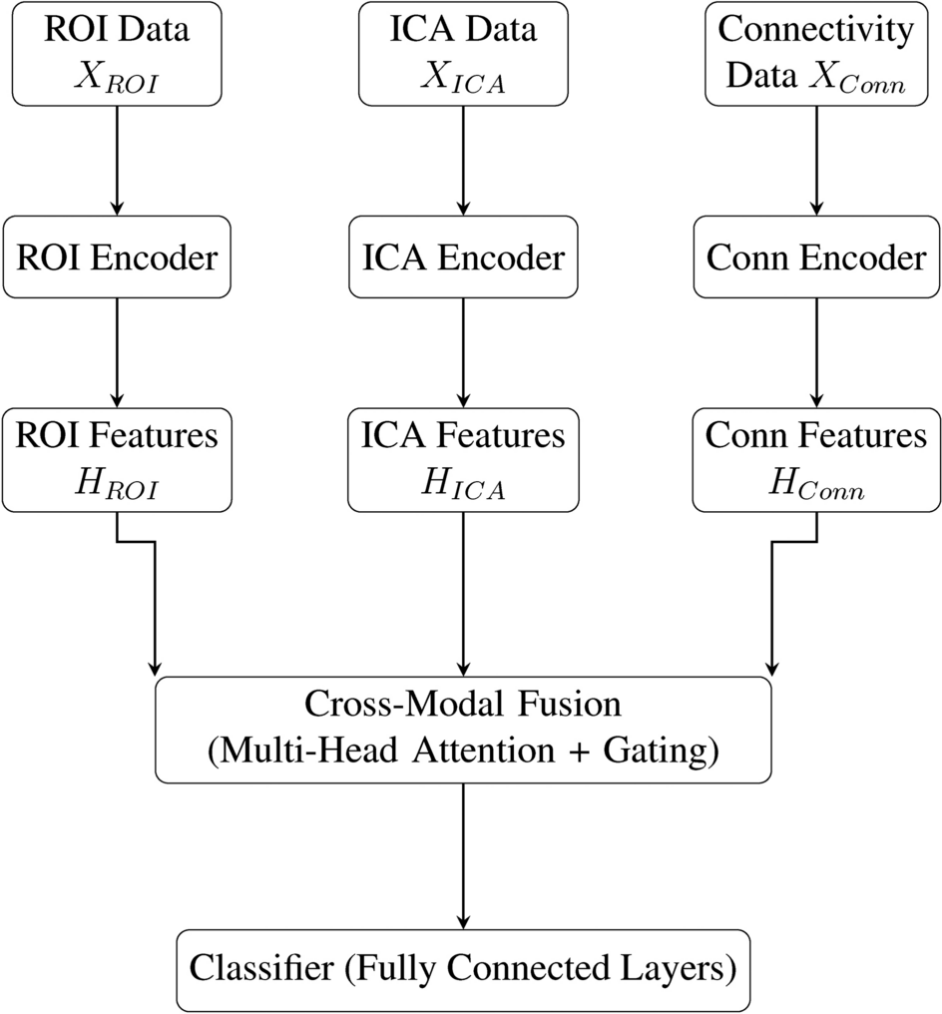
Schematic of the IMPACT framework. ROI, ICA, and connectivity features are extracted and processed by modality-specific transformers. Cross-modal fusion integrates these representations, followed by classification layers to distinguish PD from HC.

IMPACT integrates the three feature streams using a cross-modal attention fusion module as illustrated in Fig. 10. For input features $H_{ROI} \in \mathbb {R}^{T \times d}$, $H_{ICA} \in \mathbb {R}^{T \times d}$, and $H_{Conn} \in \mathbb {R}^{N_w \times d}$, where *T* is the sequence length and *d* is the feature dimension, the fusion process occurs in two stages:

First, ROI and ICA features are integrated through multi-head attention:

5 $$\begin{aligned} H_{Cross} = \text {MultiHeadAttn}(H_{ROI}, H_{ICA}, H_{ICA}) \end{aligned}$$

where the attention operation for each head *h* is computed as:

6 $$\begin{aligned} \text {Attn}_h(Q, K, V) = \text {Attention}\left( H_{ROI}W^Q_h, H_{ICA}W^K_h, H_{ICA}W^V_h\right) \end{aligned}$$

Here, $W^Q_h$, $W^K_h$, and $W^V_h$ are learnable projection matrices for query, key, and value representations respectively, and $d_k = d/N_{heads}$ is the per-head dimension.

Additionally, connectivity features are introduced via a gating mechanism, where scalar weights are learned to balance the contribution of each modality:

7 $$\begin{aligned} f_{fused} = \alpha _{ROI}H_{ROI}^{mean} + \alpha _{ICA}H_{ICA}^{mean} + \alpha _{Conn}H_{Conn}^{mean} \end{aligned}$$

where $\alpha _{ROI} + \alpha _{ICA} + \alpha _{Conn} = 1$ and $H_{m}^{mean}$ denotes temporal mean pooling of modality *m* features. The gating parameters $\{\alpha _{m}\}$ are computed through a softmax operation:

8 $$\begin{aligned} \alpha _m = \frac{\exp (W_m h_m + b_m)}{\sum _{k \in \{ROI,ICA,Conn\}} \exp (W_k h_k + b_k)} \end{aligned}$$

where $W_m$ and $b_m$ are learnable parameters specific to each modality. This formulation ensures that the fusion process maintains differentiability while providing interpretable attention weights.

The gating mechanism ensures smooth gradient flow through each modality stream via the softmax operation. Unlike hard attention or binary gates, this continuous formulation allows gradients to propagate through all paths during backpropagation, weighted by the attention scores. This helps prevent potential gradient bottlenecks that could occur if certain modalities were completely shut off during training.

The computational complexity of the fusion module is $\mathcal {O}(T^2d + Td^2)$, dominated by the attention computation and projection operations. Memory requirements scale linearly with sequence length and feature dimension:

9 $$\begin{aligned} M_{fusion} = T(d_{ROI} + d_{ICA}) + N_w d_{conn} + 2Td_h \end{aligned}$$

where $d_h$ is the hidden dimension of the fusion layer.

#### Classification layer

The fused representation $f_{fused}$ is passed through a sequence of fully connected layers with residual connections, layer normalization, and GELU activations. A final linear layer outputs logits for binary classification (PD vs. HC). Let *z* denote the output logits. The probability for class *y* is computed via a softmax function:

10 $$\begin{aligned} p(y=1|X) = \frac{\exp (z_1)}{\exp (z_0)+\exp (z_1)}. \end{aligned}$$

### Transformer architecture details

**Fig. 11:**
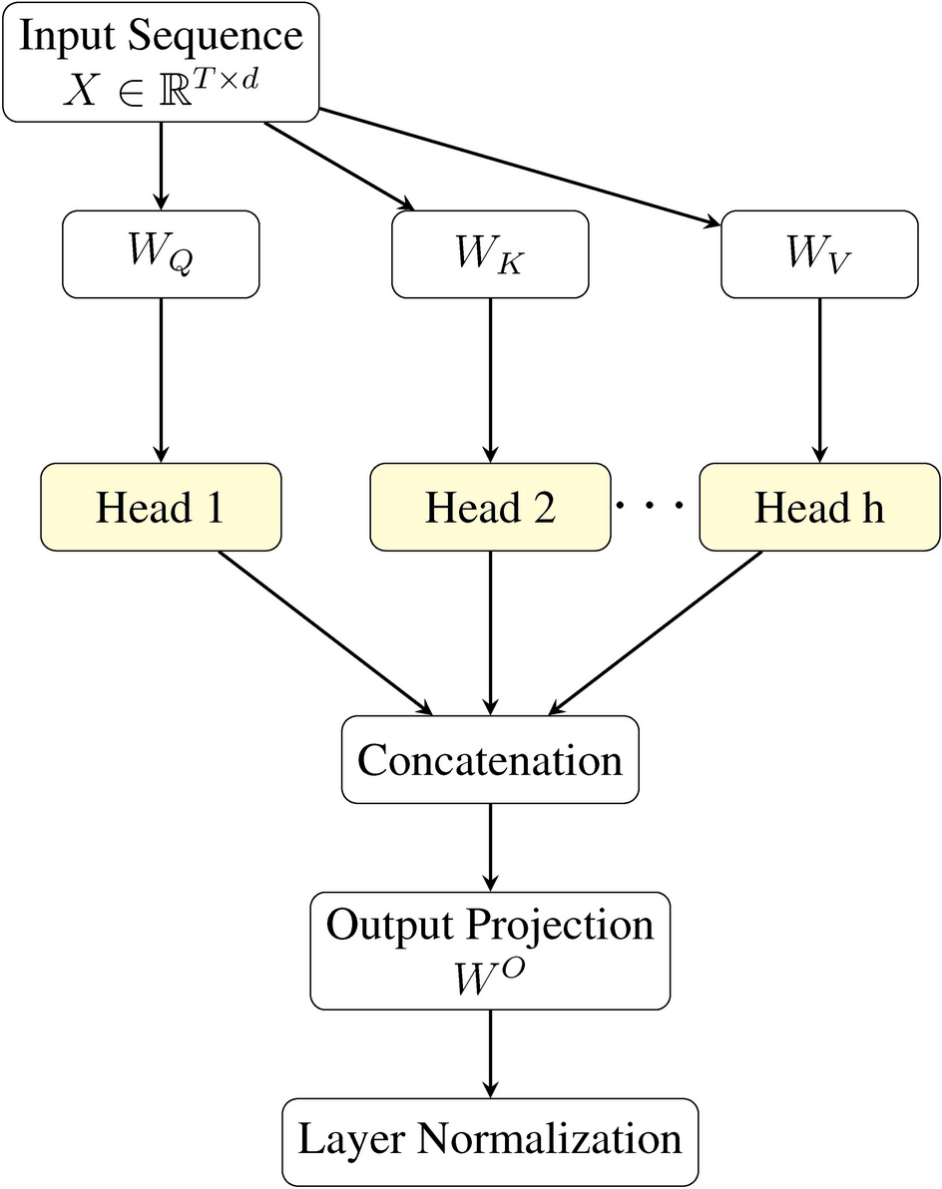
Detailed architecture of the multi-head attention mechanism. The input sequence is projected into Query (Q), Key (K), and Value (V) representations for each attention head. The outputs of all heads are concatenated and projected to the final output dimension, followed by layer normalization.

The transformer architecture employs multi-head self-attention mechanisms to process temporal sequences from each modality. The detailed architecture and interaction between components is shown in Fig. 11. For a given input sequence $X \in \mathbb {R}^{T \times d}$...

11 $$\begin{aligned} \text {Attention}(Q, K, V) = \text {softmax}\left( \frac{QK^\top }{\sqrt{d_k}}\right) V \end{aligned}$$

where *Q*, *K*, and *V* represent the Query, Key, and Value matrices respectively, derived from linear transformations of the input:

12 $$\begin{aligned} Q&= XW_Q \end{aligned}$$

13 $$\begin{aligned} K&= XW_K \end{aligned}$$

14 $$\begin{aligned} V&= XW_V \end{aligned}$$

The multi-head attention mechanism splits this computation across *h* heads:

15 $$\begin{aligned} \text {MultiHead}(X) = \text {Concat}\left( \text {head}_1,\ldots ,\text {head}_h\right) W^O \end{aligned}$$

where each head performs:

16 $$\begin{aligned} \text {head}_i = \text {Attention}\left( XW_Q^i, XW_K^i, XW_V^i\right) \end{aligned}$$

Each transformer layer combines this multi-head attention with a position-wise feed-forward network (FFN):

17 $$\begin{aligned} \text {FFN}(x) = \text {GELU}\left( xW_1 + b_1\right) W_2 + b_2 \end{aligned}$$

GELU activations are employed instead of ReLU or other alternatives due to their smoother derivatives and better performance with layer normalization in transformer architectures. The GELU nonlinearity approximates the expected transformation of inputs following dropout, providing more stable gradient flow during training while maintaining strong representational capacity.

The learnable parameters $\{W_Q, W_K, W_V, W_1, W_2\}$ are initialized using Kaiming initialization scaled by $1/\sqrt{N_{heads}}$ to maintain stable gradient flow across different network depths. Bias terms are initialized to zero following standard transformer practice. This initialization strategy helps achieve faster convergence while maintaining stable training dynamics across the deep architecture.

The complete transformer layer includes residual connections and layer normalization:

18 $$\begin{aligned} h_1&= \text {LayerNorm}\left( X + \text {MultiHead}(X)\right) \end{aligned}$$

19 $$\begin{aligned} h_2&= \text {LayerNorm}\left( h_1 + \text {FFN}(h_1)\right) \end{aligned}$$

For temporal modeling, we incorporate relative positional encodings:

20 $$\begin{aligned} \text {RelativeAttention}(Q, K, V) = \text {softmax}\left( \frac{QK^\top }{\sqrt{d_k}} + B\right) V \end{aligned}$$

where *B* represents learned relative position biases. This allows the model to capture temporal dependencies while maintaining position-aware representations.

### Ablation studies

To systematically evaluate the contribution of each input modality to classification performance, we conducted comprehensive ablation studies. These analyses involved training variants of the IMPACT model with different combinations of input modalities: (1) ROI time series only, (2) ICA components only, (3) dynamic functional connectivity matrices only, and various combinations of these modalities. All other aspects of the model architecture remained constant, ensuring that performance differences could be attributed solely to the inclusion or exclusion of specific input types.

The ablation experiments followed the same cross-validation procedure as the main model evaluation, with 5-fold cross-validation on the Neurocon dataset. For each configuration, we computed performance metrics (AUC, accuracy, balanced accuracy, F1 score, and MCC) to enable direct comparison. This approach allows for quantitative assessment of each modality’s individual contribution to the model’s discriminative power. Importantly, these ablation experiments were conducted with fewer training epochs and simplified optimization compared to the full IMPACT model reported in the main results, making them suitable for comparative analysis but not direct performance benchmarking.

When applying similar analyses to the Tao Wu dataset, which exhibited dimensionality differences between HC (45 ROIs) and PD (48 ROIs) groups, we standardized the input by using the first 45 ROIs for both groups to ensure consistent comparison. This preprocessing step demonstrates the model’s adaptability to different datasets with varying acquisition parameters.

### Model training

The model parameters are optimized using AdamW with a learning rate of $1 \times 10^{-4}$, weight decay of $5 \times 10^{-4}$, and a OneCycleLR scheduler for 150 epochs. Gradient clipping with a maximum norm of 0.1 ensures stable training. Early stopping is applied with a patience of 25 epochs (training stops if validation performance does not improve for 25 consecutive epochs). A weighted cross-entropy loss addresses class imbalance, where weights are inversely proportional to class frequencies.

Model evaluation employed leave-one-out cross-validation (LOOCV), where each subject served as the test set exactly once while the remaining subjects formed the training set. This approach explicitly prevents data leakage by ensuring all modalities (ROI time series, ICA components, and connectivity matrices) from the same participant remained together in either training or testing sets, never split across folds. While stratified k-fold cross-validation would provide more computational efficiency, LOOCV was selected for three key reasons: (1) the relatively small sample size (n=43) made computational cost less prohibitive, (2) maximal use of training data in each fold, and (3) natural handling of subject-level dependencies in longitudinal fMRI data.

#### Parameter selection and justification

The window size of 50 TR was selected based on extensive empirical testing and physiological considerations. This duration (approximately 175 s for Neurocon and 100 s for Tao Wu) captures meaningful temporal dynamics while ensuring:

1. Sufficient samples for stable correlation estimation (>30 time points recommended for reliable functional connectivity)
2. Coverage of typical resting-state fluctuation cycles (10-100 s)
3. Sensitivity to clinically relevant PD state changes (tremor episodes, cognitive fluctuations)

The choice of 5 ICA components was determined through:

1. Stability analysis showing consistent recovery of major networks (motor, default mode, executive control)
2. Cross-validation performance plateauing beyond 5 components
3. Alignment with established resting-state networks implicated in PD pathophysiology

All model parameters are summarized in Table 6.

**Table 6:** Model Architecture and Training Parameters.

Parameter Category	Value/Description
Architecture Parameters	
ROI Hidden Dimension	256
ICA Hidden Dimension	256
Number of Attention Heads	8
Number of Transformer Layers	2
Dropout Rate	0.2
Activation Function	GELU
Training Parameters	
Batch Size	16
Initial Learning Rate	1e-3
Weight Decay	1e-4
Number of Epochs	200
Early Stopping Patience	25
Loss Function	Weighted Cross-Entropy + L1 Regularization
Optimizer	AdamW
Learning Rate Scheduler	OneCycleLR
OneCycleLR Parameters	
Max Learning Rate	1e-3
Div Factor	25.0
Final Div Factor	1000.0
Pct Start	0.3
Data Processing	
Sequence Length	Variable (Dataset Dependent)
Input Normalization	Z-score per subject
Gradient Clipping	1.0

### Interpretability and visualization

#### Feature attribution

Interpretability is examined using two complementary methods:

1. **Grad-CAM:** Highlights time points and ROIs most influential for classification by computing gradients with respect to intermediate features.

2. **Attention Weight Analysis:** Examines the learned attention patterns to understand how the transformer prioritizes different temporal segments, components, and connectivity states.

#### Network and statistical analysis

Dynamic connectivity patterns are further assessed using network measures (e.g., clustering coefficients) to elucidate group-level differences. Mann-Whitney U tests and bootstrapped confidence intervals are applied to evaluate statistical significance. Multiple comparison corrections (FDR) ensure robust inference. Effect sizes (Cohen’s d) facilitate interpretation of between-group differences.

### Software and reproducibility

All data preprocessing and analyses are implemented in Python 3.8 using PyTorch (v1.10) for deep learning, FSL (v6.0) for fMRI preprocessing and ICA, scikit-learn (v0.24) for evaluation metrics, and Captum (v0.4) for interpretability. Seaborn (v0.11) and Matplotlib (v3.3) generate visualizations. Random seeds are fixed to ensure reproducibility. Model states are saved using PyTorch’s state_dict, enabling subsequent reproducibility and fine-tuning.

### Baseline models

To establish meaningful performance benchmarks, we implemented seven well-established deep learning architectures chosen for their proven capabilities in temporal sequence analysis: 2D/1D Convolutional Neural Networks (CNN) using hierarchical feature extraction for spatiotemporal pattern recognition, bidirectional LSTM and GRU networks designed to capture long-range temporal dependencies, Temporal Convolutional Networks (TCN) for capturing multi-scale temporal patterns, Multi-Layer Perceptron (MLP) as a simple baseline, and a temporal autoencoder that learns compressed representations while preserving temporal dynamics. These architectures represent distinct and complementary approaches to analyzing time-series data, each with demonstrated success in neuroimaging applications. All models process identical preprocessed input data, enabling direct performance comparisons with IMPACT.

### Methodological innovation and potential extensions

The IMPACT architecture introduces several key methodological advances in multimodal time-series analysis. First, the dual-stream approach combining raw time series with dynamic connectivity windows provides complementary views of the temporal data at different granularities. This design allows the model to capture both immediate patterns in the raw signals and evolving relationships between features, addressing a common challenge in temporal machine learning where important information may exist at multiple time scales.

Second, the gating and attention mechanism prevents the common problem of modality overshadowing, where stronger signals might dominate the model’s decisions even when weaker signals carry critical information. The gating layer adaptively weights each modality’s contribution based on its time-varying relevance, while the attention mechanism identifies important temporal segments within each modality. This synergistic combination allows the model to maintain sensitivity to subtle patterns while still using strong signals when appropriate.

The architecture’s inductive bias favors the discovery of correlated, time-varying signals across modalities—a property particularly valuable in domains beyond neuroimaging where parallel data streams may exhibit complex temporal relationships. Potential extensions of this approach include:

- Handling missing modalities through learned interpolation or robust fusion mechanisms
- Real-time updating of attention weights for streaming applications
- Adaptive window sizing based on detected pattern frequencies
- Integration with domain-specific priors through structured attention constraints

These extensions would further enhance the model’s utility across diverse application domains while maintaining its core strengths in temporal pattern detection and interpretability.

### Model comparison and statistical analysis

Statistical comparisons between IMPACT and baseline models were conducted using non-parametric Mann-Whitney U tests due to the relatively small sample sizes and potential non-normality of performance metrics. Effect sizes were quantified using Cliff’s delta, and 95% confidence intervals were computed using bootstrapping with 10,000 resamples. To account for multiple comparisons when testing IMPACT against each baseline model, p-values were adjusted using the Benjamini-Hochberg procedure with a false discovery rate $\le$ 0.05.

## Supplementary Information

Supplementary Information.

## Data Availability

The complete implementation of IMPACT is available as an open-source project at https://github.com/salilp42/IMPACT. The neuroimaging data used in this study is from the Neurocon and Tao Wu datasets, which are both publicly available through the NITRC platform at https://fcon_1000.projects.nitrc.org/indi/retro/parkinsons.html.
