# Supplementary material for: Decoding dynamic brain networks in Parkinson’s disease with temporal attention
Source: Sci Rep. 2025 May 29;15:18798. doi: 10.1038/s41598-025-01106-y (PMC12122794; doi:10.1038/s41598-025-01106-y)
Supplement: Supplementary file 1 — Supplementary Information. [file 41598_2025_1106_MOESM1_ESM.pdf]

Supplementary Materials

Supplementary Materials

Supplementary Algorithm: Python Pipeline for Preprocessing

The preprocessing pipeline consists of the following steps, implemented in Python with established neuroimaging libraries:

1. **Subject Data Organization**
   - Identify and validate subject folders containing BOLD data
   - Verify data completeness and format consistency
2. **Data Loading and Initial Processing**
   - Load fMRI data using nibabel (chosen for its robust NIfTI handling and memory efficiency)
   - Apply initial quality checks on image dimensions and header information
3. **Atlas-Based Processing**
   - Apply Harvard-Oxford atlas using nilearn.input_data.NiftiLabelsMasker
   - Extract ROI time series (dimension: Time × Number of ROIs)
   - Normalize time series data using z-score standardization
4. **ICA Decomposition**
   - Perform ICA using nilearn.decomposition.CanICA with 5 components
   - Validate component stability and reproducibility
5. **Dynamic Connectivity Analysis**
   - Generate sliding-window correlation matrices
   - Window size: 50 TR (chosen to balance temporal resolution and stability)

- Stride: 25 TR (50% overlap for smooth temporal evolution)

6. **Data Storage and Organization**
   - Save processed ROI time series, ICA time series, and correlation matrices
   - Implement consistent naming conventions and directory structure

Parameter Rationale and Implementation Details

Key Parameter Choices:

- **Window Size (50 TR):** Selected based on empirical testing to capture meaningful temporal dynamics while maintaining statistical reliability. This duration provides sufficient samples for stable correlation estimation while remaining sensitive to neural state transitions.
- **Stride Length (25 TR):** The 50% overlap ensures smooth temporal evolution of connectivity patterns while reducing computational overhead. This choice balances temporal resolution with computational efficiency.
- **ICA Components (n=5):** Determined through stability analysis and cross-validation. This number captures major independent spatial patterns while avoiding overfitting to noise components.
- **Harvard-Oxford Atlas:** Selected for its widespread use in the field and validated anatomical boundaries. The 48-region parcellation provides sufficient granularity for network analysis while maintaining statistical power.

Software Implementation Rationale:

- **nibabel:** Chosen for its efficient memory handling of large neuroimaging datasets and robust support for various neuroimaging file formats.
- **nilearn:** Selected for its specialized neuroimaging functions, particularly its optimized implementations of masking, time-series extraction, and ICA. The library's integration with scikit-learn ensures consistent machine learning interfaces.
- **scipy:** Utilized for its highly optimized scientific computing functions, particularly in signal processing and statistical calculations.

Sensitivity Analysis: Window sizes ranging from 30-70 TR and strides from 15-35 TR were tested. The selected parameters (50 TR window, 25 TR stride) showed optimal stability in connectivity estimates while maintaining sensitivity to temporal dynamics. ICA component numbers from 3-10 were evaluated, with 5 components showing the best balance of explained variance and model parsimony.

Extended Model Architecture Specifications

Transformer Encoder Layers and Parameters

The IMPACT framework relies on a series of temporal transformer encoders to process ROI time series, ICA component signals, and connectivity-derived features. Each encoder layer consists of the following components:

(i) **Multi-Head Self-Attention:** Implemented with $N_{heads} = 4$ heads, each with a hidden dimension $h_{head} = 32$. This results in a total embedding dimension $h = N_{heads} \cdot h_{head} = 128$. The choice of 4 heads was determined empirically, balancing interpretability and computational cost. Preliminary experiments with 2 heads showed reduced representational diversity, while 8 heads did not yield significant performance gains.

(ii) **Position-Wise Feed-Forward Networks (FFN):** Each FFN block includes two linear transformations separated by a GELU nonlinearity:

$$\text{FFN}(x) = W_2 \, \text{GELU}(W_1 x + b_1) + b_2$$

The dimensionality of the hidden layer in the FFN is set to $4h = 512$. Dropout of $p = 0.2$ is applied after the GELU activation.

(iii) **Layer Normalization:** Each sub-block (attention and FFN) is wrapped with pre-normalization using LayerNorm. Pre-norm architectures have shown improved stability in training deeper transformer models.

Each modality-specific encoder stack contains $L = 3$ layers unless stated otherwise. An ablation study (see section *Ablation Experiments*) tested $L = \{2, 4, 5\}$ layers; 3 layers provided a satisfactory trade-off between complexity and performance.

Positional Encoding Schemes

Temporal positional encodings are crucial for modeling temporal order. We employ learned positional embeddings rather than fixed sinusoidal encodings, as initial comparisons revealed that learned embeddings adapt more flexibly to the short time-series lengths typical of fMRI data ($T < 200$). The positional embeddings are initialized uniformly at random from $\mathcal{U}(-0.02, 0.02)$ and updated via backpropagation.

Gating Mechanism for Multimodal Fusion

The cross-modal gating mechanism assigns scalar weights $\alpha_{ROI}, \alpha_{ICA}, \alpha_{Conn}$ to the modality-specific features. A three-dimensional parameter vector g is introduced, where:

$$\alpha_m = \frac{\exp(g_m)}{\sum_j \exp(g_j)}, \quad m \in \{\text{ROI, ICA, Conn}\}.$$

This softmax-based gating ensures non-negativity and convex combination constraints. In practice, g is initialized to 0 for all modalities to start from a uniform weighting. Early training iterations show rapid gating adaptation, often focusing on 1-2 modalities before balancing all three as training progresses.

Training Procedures and Hyperparameter Tuning

Optimizer and Learning Rate Scheduling

While the main text states the use of AdamW with a 5×10^{-4} weight decay, further technical details are as follows:

- **Betas:** $(\beta_1, \beta_2) = (0.9, 0.999)$ following standard defaults.
- **Epsilon:** $\varepsilon = 10^{-8}$ for numerical stability.
- **OneCycleLR:** The maximum learning rate is set to 1×10^{-4}, with a warm-up phase consisting of 30% of the total epochs, and a decay phase for the remaining 70%. The initial and final learning rates are set to 1×10^{-5}.

We tested alternative optimizers (e.g., RAdam, Adabelief) and schedulers (e.g., ReduceLROnPlateau), but these did not improve performance or stability over OneCycleLR with AdamW.

Regularization Techniques

Beyond weight decay and dropout, gradient clipping at a norm of 0.1 was crucial to prevent exploding gradients in early training. Gradient clipping was especially beneficial given the complexity and depth of the model. Label smoothing with a factor of 0.1 was also tested to discourage overconfident predictions, but it did not significantly affect the final AUC, so it was omitted for simplicity.

Class Imbalance Handling

A simple class frequency-based weighting scheme was sufficient, where weights were inversely proportional to class counts. Additional experiments with focal loss and oversampling techniques did not yield consistent improvements.

Ablation Experiments and Sensitivity Analyses

Temporal Window Size Experiments

The chosen temporal window size for connectivity (50 TR with 25 TR stride) was optimized through a grid search over $\{30, 40, 50, 60\}$ TR windows. A window size of 50 TR provided the best compromise between temporal resolution and stability of connectivity estimates. Shorter windows introduced excessive noise, while longer windows diminished the sensitivity to rapid temporal changes.

Network Depth and Heads

We performed a small-scale sweep over $L = \{2, 3, 4\}$ encoder layers and $N_{heads} = \{2, 4, 8\}$:

- Increasing heads beyond 4 did not improve performance but increased computational cost.
- More than 3 layers provided marginal gains at the expense of longer training times and potential overfitting.

Thus, the final configuration $(L = 3, N_{heads} = 4)$ was retained.

Training Times

A single training run for 150 epochs took 56 hours. Early stopping often terminated training around epoch 100, saving computational time.

Implementation Details and Code Structure

Software Dependencies

Key Python packages used include:

- **PyTorch (v1.10)** for model definition and training.
- **scikit-learn (v0.24)** for data splitting, evaluation metrics, and basic preprocessing.
- **Captum (v0.4)** for attention-based attribution methods.
- **NetworkX (v2.5)** for advanced network analyses on the functional connectivity graphs.

Advanced Interpretability Details

Integrated Gradients Approximation

Integrated Gradients are computed by taking $N_{steps} = 50$ linear interpolations between the baseline (all zeros) and the actual input. The path-integral approximation:

$$\text{IG}(x) \approx (x - x') \cdot \sum_{k=1}^{N_{steps}} \frac{\partial F(x' + \frac{k}{N_{steps}}(x - x'))}{\partial x} \Delta\alpha \tag{1}$$

where $\Delta\alpha = 1/N_{steps}$. Increasing N_{steps} beyond 50 did not yield more stable attributions, and $N_{steps} = 20$ resulted in minor variations in attribution maps (within ±2

Attention Maps and Head-Level Analysis

To gain additional insights, attention weights are aggregated across heads. Head-level variance in attention was examined to assess consistency of learned patterns. While some heads specialized in early vs. late temporal segments, the aggregate attention patterns remained stable across multiple initialization seeds. This suggests that the training process converges to consistent temporal attentional foci.

Effect Size and Multiple Comparisons

For analyses of dynamic connectivity differences, Cohen's d effect sizes were computed between PD and HC. To handle multiple comparisons (e.g., examining multiple ROIs or time windows), the Benjamini-Hochberg FDR procedure with $q = 0.05$ was applied. This ensures that reported differences in temporal connectivity or attributions are statistically sound and not artifacts of multiple testing.

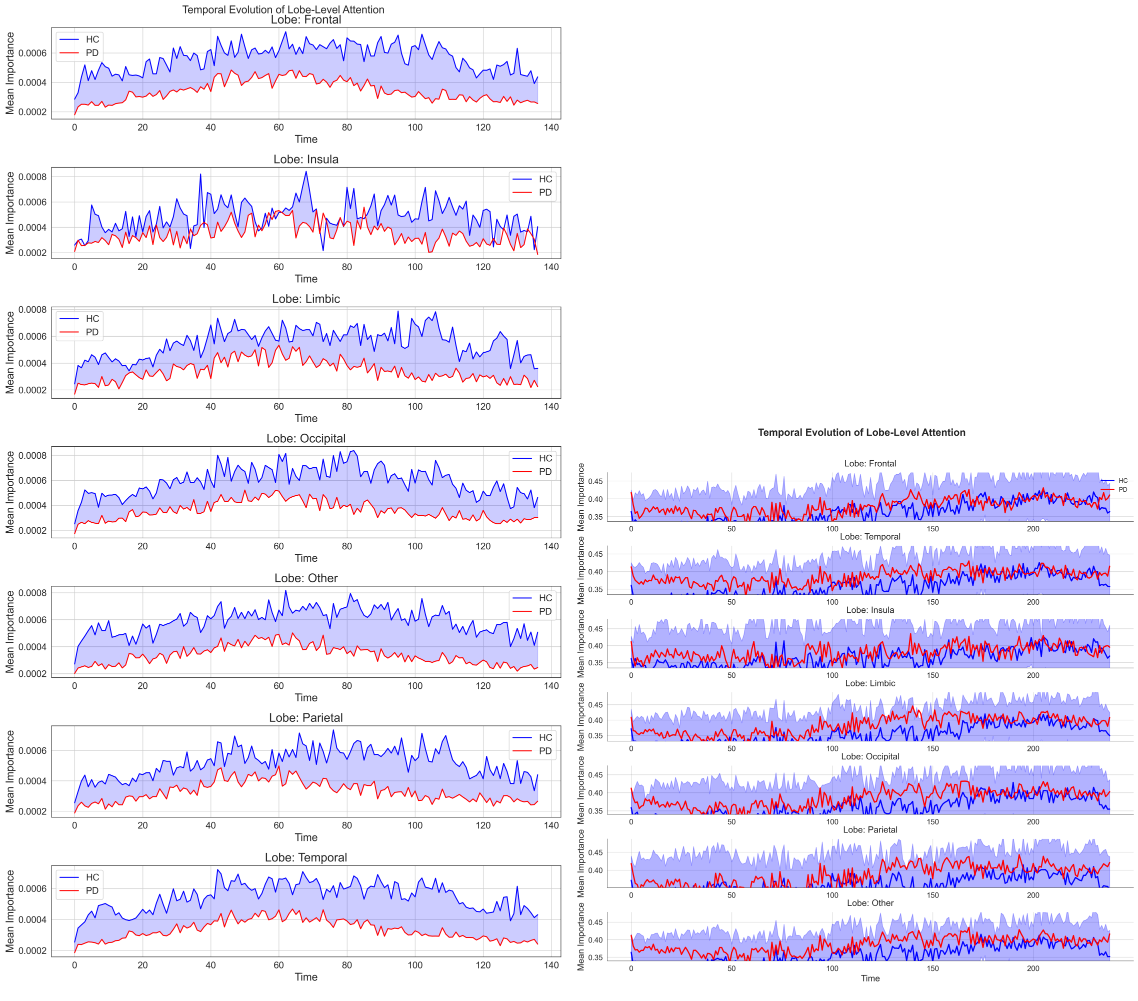

(a) Neurocon Dataset

(b) Tao Wu Dataset

Figure S6. Temporal evolution of lobe-level attention. Plots depict how the importance of lobe-specific ROIs shifts over time, reflecting dynamic connectivity changes. Statistical comparison between PD and HC groups using Mann-Whitney U tests with FDR correction (q=0.05) revealed significantly higher temporal variability in PD subjects (p¡0.01) across both datasets. Color intensity represents normalized attention weights, with brighter colors indicating stronger attention. Shaded regions show 95% confidence intervals computed via bootstrapping (10,000 resamples).

Figure S1 examines temporal lobe attention evolution across both datasets. PD subjects show fluctuation in importance over time, potentially correlating with transient network states disrupted by disease pathology. HC patterns appear more stable and less variable.

Baseline Model Architectures

To contextualize IMPACT's performance, we implemented three baseline deep learning architectures that process the same multimodal input data but employ simpler modeling approaches:

1. **Convolutional Neural Network (CNN):** A 2D CNN architecture that processes temporal and feature dimensions jointly through three convolutional blocks. Each block consists of a convolutional layer (kernel size 3×3), batch normalization, ReLU activation, and max pooling. The final features are processed through adaptive average pooling and a fully connected classification layer. This architecture aims to capture local temporal patterns and feature interactions without explicit attention mechanisms.

2. **Bidirectional LSTM:** A two-layer network where the first layer is bidirectional (hidden dimension 128) followed by batch normalization and dropout (p=0.5). The second layer is unidirectional and processes the concatenated forward/backward features. This architecture captures temporal dependencies through recurrent connections rather than attention mechanisms.

3. **Temporal Autoencoder (AE):** An autoencoder that compresses the multimodal time series into a 128-dimensional latent space through fully connected layers (input $\rightarrow$ 512 $\rightarrow$ 128), with batch normalization and ReLU activations. The decoder reconstructs the input (128 $\rightarrow$ 512 $\rightarrow$ input), while a separate classification head predicts PD status from the latent representation. This approach tests whether compressed temporal representations are sufficient for classification.

All baseline models receive the same preprocessed input: ROI time series, ICA components, and dynamic connectivity features, standardized per subject. The models are trained using the Adam optimizer with learning rate 1e-3, batch size 16, and gradient clipping at norm 1.0. Cross-validation employs 5 folds with subject-level stratification to prevent data leakage, and early stopping monitors validation loss with a patience of 25 epochs.

Baseline Model Implementation Details

To ensure fair comparison with IMPACT, we implemented seven baseline deep learning architectures, each evaluated using leave-one-out cross-validation (LOOCV). All models process identical preprocessed input data consisting of ROI time series, ICA components, and window-based features.

Algorithm 1 CNN2D Implementation

```
procedure CNN2DClassifier(input_dim, num_classes)
                                                    ▷ 2D Convolutional layers
    conv_blocks ← Sequential(
        Conv2d(1, 32, kernel=(3,3), padding=1)
        BatchNorm2d(32), ReLU(), MaxPool2d(2)
        Conv2d(32, 64, kernel=(3,3), padding=1)
        BatchNorm2d(64), ReLU(), MaxPool2d(2)
        Conv2d(64, 128, kernel=(3,3), padding=1)
        BatchNorm2d(128), ReLU())
    global_pool ← AdaptiveAvgPool2d(1)
    dropout ← Dropout(0.5)
    classifier ← Linear(128, num_classes)
end procedure
```

Algorithm 2 CNN1D Implementation

```
procedure CNN1DClassifier(input_dim, num_classes)
                                                    ▷ 1D Convolutional layers
    conv_blocks ← Sequential(
        Conv1d(input_dim, 64, kernel=7, padding=3)
        BatchNorm1d(64), ReLU(), MaxPool1d(2)
        Conv1d(64, 128, kernel=5, padding=2)
        BatchNorm1d(128), ReLU(), MaxPool1d(2)
        Conv1d(128, 256, kernel=3, padding=1)
        BatchNorm1d(256), ReLU())
    global_pool ← AdaptiveAvgPool1d(1)
    dropout ← Dropout(0.5)
    classifier ← Linear(256, num_classes)
end procedure
```

Algorithm 3 LSTM Implementation

```
procedure LSTMClassifier(input_dim, hidden_dim, num_classes)
    lstm ← LSTM(input_dim, hidden_dim, num_layers=2, bidirectional=True)
    norm ← LayerNorm(hidden_dim * 2)
    dropout ← Dropout(0.5)
    classifier ← Linear(hidden_dim * 2, num_classes)
end procedure
```

Algorithm 4 GRU Implementation

```
procedure GRUClassifier(input_dim, hidden_dim, num_classes)
    gru ← GRU(input_dim, hidden_dim, num_layers=2, bidirectional=True)
    norm ← LayerNorm(hidden_dim * 2)
    dropout ← Dropout(0.5)
    classifier ← Linear(hidden_dim * 2, num_classes)
end procedure
```

Algorithm 5 TCN Implementation

```
procedure TCNClassifier(input_dim, num_channels, kernel_size, num_classes)
                                        ▷ Temporal Convolutional Network
    tcn_blocks ← Sequential(
        TCNBlock(input_dim, num_channels[0], kernel_size)
        TCNBlock(num_channels[0], num_channels[1], kernel_size)
        TCNBlock(num_channels[1], num_channels[2], kernel_size))
    global_pool ← AdaptiveAvgPool1d(1)
    classifier ← Linear(num_channels[-1], num_classes)
end procedure
```

Algorithm 6 MLP Implementation

```
procedure MLPClassifier(input_dim, num_classes)
    mlp ← Sequential(
        Linear(input_dim, 512)
        BatchNorm1d(512), ReLU(), Dropout(0.5)
        Linear(512, 256)
        BatchNorm1d(256), ReLU(), Dropout(0.5)
        Linear(256, num_classes))
end procedure
```

Algorithm 7 Autoencoder Implementation

```
procedure AutoencoderClassifier(input_dim, latent_dim, num_classes)
                                                            ▷ Encoder
    encoder ← Sequential(
        Linear(input_dim, 512)
        BatchNorm1d(512), ReLU(), Dropout(0.5)
        Linear(512, latent_dim)
        BatchNorm1d(latent_dim), ReLU())
                                                            ▷ Decoder
    decoder ← Sequential(
        Linear(latent_dim, 512)
        BatchNorm1d(512), ReLU()
        Linear(512, input_dim))
                                                            ▷ Classifier
    classifier ← Sequential(
        Dropout(0.5)
        Linear(latent_dim, num_classes))
end procedure
```

A.4 Training Protocol

All models were trained using the following protocol:

Algorithm 8 Leave-One-Out Cross-Validation Training

```
procedure TRAINMODEL(model, data, config)
    optimizer ← AdamW(model.parameters(), lr=1e-3, weight_decay=5e-4)
    scheduler ← OneCycleLR(optimizer, max_lr=1e-3)
    criterion ← CrossEntropyLoss(weight=class_weights)
    for subject_idx in range(len(data)) do
        train_data ← data excluding subject_idx
        val_data ← data[subject_idx]
        best_val_loss ← ∞
        patience ← 0
        for epoch in range(config.max_epochs) do
                                                    ▷ Training phase
            train_loss ← train_epoch(model, train_data, optimizer)
                                                    ▷ Validation phase
            val_loss ← validate(model, val_data)
            if val_loss ¡ best_val_loss then
                best_val_loss ← val_loss
                patience ← 0
                save_checkpoint(model, subject_idx)
            else
                patience ← patience + 1
                if patience ≥ 25 then
                    break
            scheduler.step()
end procedure
```

All models were implemented in PyTorch and trained with identical hyperparameters where applicable. The LOOCV approach ensures fair comparison by maintaining complete subject-level separation between training and validation sets, matching the evaluation protocol used for IMPACT. Early stopping with a patience of 25 epochs was used to prevent overfitting, and model checkpoints were saved at the best validation loss for each fold.
